# Maternal Stress Primes Adolescent Stress Susceptibility via Microglial Complement-Dependent Synaptic Phagocytosis

**DOI:** 10.34133/research.1367

**Published:** 2026-08-05

**Authors:** Xiaoqing Wang, Ming Jiang, Ling Wei, Xinyue Liang, Fang Fang, Yunli Xie, Jing Cang

**Affiliations:** ^1^ Department of Pain Management, Shanghai Geriatric Medical Center, Zhongshan Hospital, Fudan University, Shanghai 201104, China.; ^2^ Department of Pain Management, Zhongshan Hospital, Fudan University, Shanghai 200032, China.; ^3^ Department of Anesthesiology, Zhongshan Hospital, Fudan University, Shanghai 200032, China.; ^4^ State Key Laboratory of Medical Neurobiology and MOE Frontiers Center for Brain Science, Institutes of Brain Science, Fudan University, Shanghai, China.

## Abstract

Early-life adversity, including prenatal stress exposure, has enduring effects on stress responsivity later in life. Yet the impact of maternal stress on offspring susceptibility to stress and its underlying biological mechanisms remain to be elucidated. Here, we established a “2-hit” stress model to investigate whether maternal chronic unpredictable stress (E9.5 to E18) increases offspring susceptibility to social isolation during adolescence (P28 to P49). Our study reveals that maternal stress increases susceptibility to adolescent social isolation in male—but not female—offspring, manifested as increased anxiety- and depressive-like behaviors. This vulnerability is mediated by the priming of hippocampal dentate gyrus microglia through the complement C3–C3aR signaling pathway, which promotes the aberrant phagocytosis of excitatory synapses and reduces glutamatergic neuronal activity. Notably, pharmacological blockade of C3aR or chemogenetic activation of glutamatergic neurons in the dentate gyrus during adolescence effectively alleviated stress susceptibility. Therefore, our findings identify a targetable immune-mediated mechanism in the hippocampus that underlies stress vulnerability in offspring exposed to gestational maternal stress, offering new avenues for preventing adolescent-onset mood disorders.

## Introduction

Adolescence represents a critical neurodevelopmental transition marked by profound reorganization of brain circuitry, emotional regulation, and social cognition [1,2]. During this period, individuals encounter diverse psychosocial stressors, including academic demands, peer competition, and evolving social hierarchies. While many adolescents demonstrate resilience in navigating these challenges, a substantial proportion exhibit heightened vulnerability to stress-related mood disorders, particularly depression [3–6]. This inter-individual variability in stress susceptibility is thought to reflect underlying differences in neurobiological programming, yet the mechanisms governing this differential vulnerability remain incompletely understood. Given the rising global prevalence of adolescent depression and its profound societal burden, elucidating the neurobiological substrates of stress vulnerability has become an urgent priority.

The “2-hit” hypothesis offers a tractable framework for dissecting this question, proposing that early-life adversity (the first hit) induces latent neurobiological alterations that shape individuals’ responses to subsequent challenges (the second hit) encountered later in life [7–10]. The prenatal period is particularly relevant in this context, as the foundational architecture of the nervous system is laid down during gestation through tightly orchestrated programs of neurogenesis, neuronal migration, and synaptic assembly [11–15]. Notably, psychological distress during pregnancy is remarkably prevalent: the World Health Organization estimates that approximately 10% of pregnant women worldwide experience mental health disorders, with rates reaching 15.6% in low- and middle-income countries [16]. Maternal stress has been causally linked to fetal hypothalamic–pituitary–adrenal (HPA) axis dysregulation, epigenetic reprogramming, and perturbation of the neuroimmune microenvironment [17,18], disruptions that may fundamentally alter trajectories of brain development. Because adolescence itself constitutes a second sensitive window of synaptic remodeling and heightened plasticity, prenatal perturbations may preconfigure an individual’s stress-response phenotype during this later stage. Nevertheless, the cellular and molecular mechanisms underpinning these effects remain to be fully elucidated.

Among brain regions implicated in stress reactivity, the hippocampus—and the dentate gyrus (DG) in particular—has emerged as a critical node of stress susceptibility. Hippocampal volume reductions in major depressive disorders are most pronounced in the DG, paralleled by postmortem losses in granule cell number and dendritic complexity [19,20]. Unlike CA1 and CA3, which achieve mature connectivity by weaning, DG granule cells continue synaptic remodeling throughout adolescence, leaving their excitatory connectivity uniquely open to activity-dependent reshaping. Functionally, the DG transforms entorhinal inputs into sparse, decorrelated representations through pattern separation, a computation exquisitely sensitive to glutamatergic drive onto granule cells [21–23]. This convergence of adolescent-extended plasticity, stress sensitivity, and glutamatergic dependence positions the DG as a plausible locus at which a prenatal “first hit” may be latently encoded and subsequently unmasked by adolescent stress. We therefore hypothesize that the DG constitutes a key anatomical node linking early-life adversity to adolescent stress susceptibility.

As the brain’s resident immune cells, microglia perform indispensable roles in circuit refinement throughout life [24–27], and their developmental trajectory is acutely sensitive to maternal perturbations during pregnancy, including immune activation, sleep disruption, and metabolic stress [28–30]. During critical developmental windows, microglia actively survey synaptic elements through highly motile processes, executing activity-dependent synaptic pruning to sculpt nascent circuits and optimize network connectivity. This microglia-mediated refinement is essential for the maturation of higher-order functions, including social cognition and emotional regulation. Critically, dysregulation of this pruning process—manifesting as either excessive or insufficient synapse elimination—disrupts the delicate excitatory/inhibitory (E/I) balance, a synaptic pathology increasingly recognized as a convergent feature of stress-related neuropsychiatric disorders, including depression and schizophrenia [31,32]. Yet whether microglia–synapse interactions mediate the impact of maternal stress on adolescent offspring stress reactivity remains to be elucidated.

At the molecular level, microglial engulfment of synapses is executed largely through the classical complement cascade. C1q secreted by microglia is deposited onto synaptic surfaces, where it triggers the cleavage of C3 into C3a and C3b. Synapses tagged with C3b/iC3b are then recognized by the microglial complement receptor 3 (CR3) and selectively eliminated, whereas the released anaphylatoxin C3a acts on its microglial receptor C3aR to promote chemotaxis and proinflammatory activation. Synaptic activity is inversely correlated with complement deposition, such that weaker synapses are preferentially marked for removal, and genetic deletion of C1q or C3 in the developing mouse retinogeniculate system causes pronounced pruning deficits and aberrant connectivity, establishing the complement system as a core executor of developmental synapse elimination [33–35]. Beyond healthy development, aberrant reactivation of this cascade has been implicated in pathological synapse loss in neurodegenerative and neuroinflammatory contexts. Yet whether the complement-driven microglial synaptic engulfment mediates the effect of maternal stress on adolescent stress susceptibility has not been established.

In this study, we employed a “2-hit” paradigm combining prenatal chronic unpredictable stress (E9.5 to E18) with adolescent social isolation (postnatal days [PNDs] 28 to 49) to model the developmental trajectory from early adversity to adolescent psychopathology. The adolescent window examined in our study (PNDs 28 to 49 in mice) corresponds approximately to early-to-middle adolescence in humans (ages 12 to 17 years), a critical neurodevelopmental period. We demonstrate that maternal stress selectively increases stress vulnerability in male offspring through a mechanism involving complement C3–C3aR-mediated microglial priming in the hippocampal DG region. This priming state predisposes microglia to execute excessive phagocytosis of excitatory synapses upon subsequent adolescent stress exposure, resulting in reduced glutamatergic neuronal excitability and depression-like behaviors. Critically, we show that this pathological cascade is reversible: either pharmacological C3aR antagonism or chemogenetic activation of DG glutamatergic neurons during adolescence largely rescues stress-induced deficits. These findings establish a causal microglia–synapse mechanism by which maternal stress programs adolescent vulnerability and identify the adolescent period as a therapeutic window for intervention to prevent stress-related mood disorders.

## Results

### Maternal stress enhances susceptibility to stress in adolescent male offspring

**Fig. 1.**
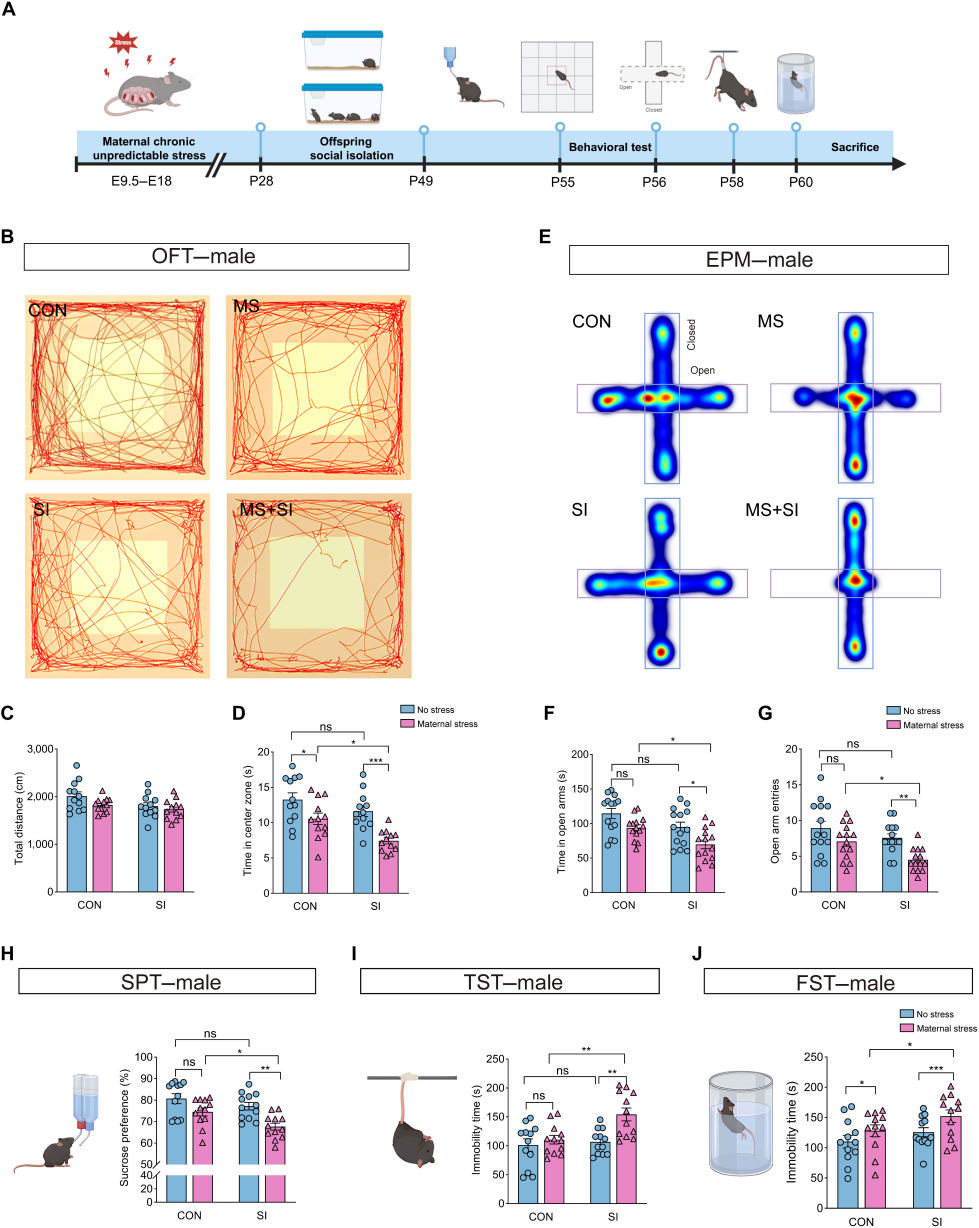
Maternal stress enhances susceptibility to stress in adolescent male offspring. (A) Schematic diagram of experimental paradigms and behavioral tests. (B) Representative traces of open-field test. (C) The total distance in the open-field test (*n* = 12). (D) The time spent in the central zone in the open-field test (*n* = 12). (E) Representative traces of the elevated plus maze. (F) The time spent in the open arms in the elevated plus maze test (*n* = 14). (G) The number of entries into the open arms in the elevated plus maze test (*n* = 14). (H) The sucrose preference ratio in the sucrose preference test (*n* = 12). (I) The immobility time in the tail suspension test (*n* = 12). (J) The immobility time in the forced swim test (*n* = 12). Quantitative results are presented as mean ± standard error of the mean (SEM). Statistical significance: *P < 0.05; **P < 0.01; ***P < 0.001; ns indicates no significant difference. Statistical comparisons were made using one-way analysis of variance (ANOVA) followed by Tukey’s post hoc analysis. CON, control group; MS, maternal stress group; SI, social isolation group; MS+SI, maternal stress + social isolation group.

To examine whether maternal stress increases stress susceptibility during adolescence, we employed a “2-hit” paradigm in which offspring were exposed to maternal stress during gestation and subsequently subjected to 3 weeks of social isolation during adolescence (Fig. 1A). A series of standardized behavioral assessments were performed to evaluate anxiety- and depression-like phenotypes, with initial analyses focusing on specifically male offspring. In the open-field test (OFT), locomotor activity, measured by total distance traveled, did not differ between groups. Compared to the control (CON) group, maternal stress (MS) mice exhibited a substantial reduction in time spent in the central area, whereas the social isolation (SI) group showed no such difference. Remarkably, the maternal stress + social isolation (MS+SI) group displayed an even shorter duration in the center than either the MS or SI group alone (Fig. 1B to D). Consistent with this, in the elevated plus maze (EPM), open-arm exploration (both time spent and entries) was significantly decreased only in MS+SI mice, but not in either MS or SI mice (Fig. 1E to G). Similar patterns were observed in depression-related behavioral tests. In the sucrose preference test (SPT), MS+SI mice displayed a marked decrease in sucrose preference, a measure of reward sensitivity and anhedonia-like behavior, whereas neither MS nor SI mice alone showed an effect (Fig. 1H). In the tail suspension test (TST), immobility time was significantly increased in MS+SI mice compared with that in MS mice or SI mice alone, whereas MS or SI alone produced no significant change (Fig. 1I). In the forced swim test (FST), MS mice exhibited increased immobility relative to CON mice, and this effect was further exacerbated in MS+SI mice compared with that in MS mice or SI mice alone (Fig. 1J). Collectively, social isolation alone did not induce significant anxiety- or depression-like behaviors. By contrast, male offspring exposed to maternal stress were sensitized to subsequent social isolation, resulting in exacerbated anxiety- and depression-like behaviors.

Given the significant effects observed in male offspring, we next investigated whether maternal stress exerted a comparable influence in females. To address this, we conducted the same battery of behavioral assessments (Fig. S1A). In the OFT, all groups exhibited comparable locomotor activity and center exploration (Fig. S1B to D). Similarly, in the EPM, both open-arm entries and time spent in the open arms were unaffected by MS, SI, or MS+SI (Fig. S1E to G). Consistent with these findings, no differences between groups were observed in the SPT or in immobility time during the TST and FST (Fig. S1H to J). Collectively, these results indicate that neither maternal stress nor social isolation, alone or in combination, influenced anxiety- or depression-like behaviors in females. Given that maternal-stress-induced susceptibility to adolescent stress was specific to male offspring, subsequent mechanistic studies were conducted exclusively in males.

### Reduced DG neuronal excitability in offspring exposed to maternal stress contributes to stress vulnerability

**Fig. 2.**
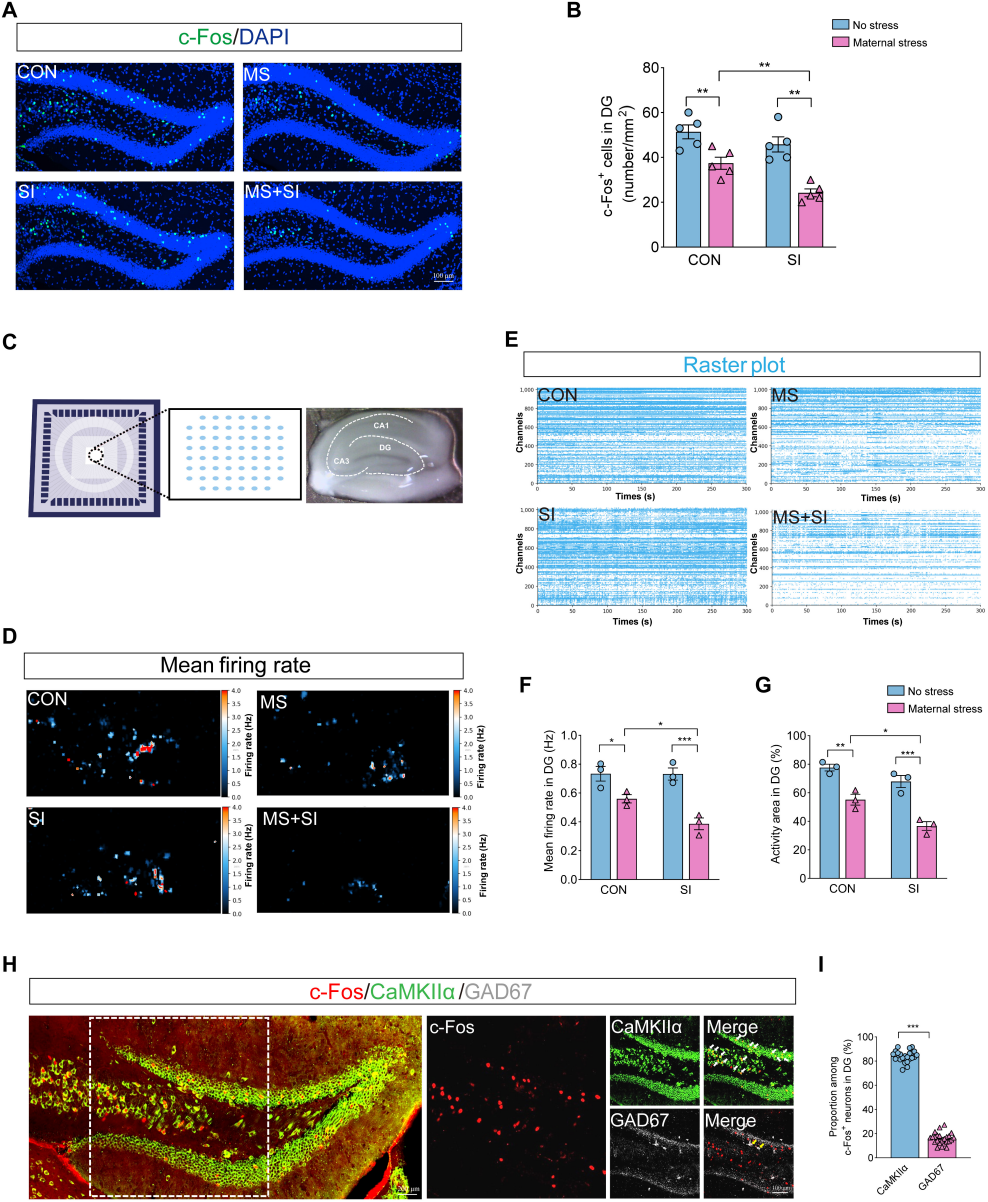
Hippocampal dentate gyrus (DG) neuronal activity reduction underlies stress sensitivity in adolescent male offspring following maternal stress. (A) Representative image of c-Fos immunofluorescence staining in the hippocampal DG region. (B) The number of c-Fos-positive cells in the hippocampal DG region. Data were collected from 5 mice per group, with 3 brain slices per mouse. (C) Schematic diagram of the multielectrode array (MEA) experiment. (D) Representative heatmap of the average spontaneous firing frequency of neurons in the hippocampal DG region. (E) Representative dot plot of the time-dependent firing activity of neurons in the hippocampal DG region. (F) Average firing frequency of neurons in the hippocampal DG region. Data were collected from 3 mice per group, with 3 brain slices per mouse. (G) Percentage of neuronal firing activity area in the hippocampal DG region. Data were collected from 3 mice per group, with 3 brain slices per mouse. (H) Representative immunofluorescence image of c-Fos, the excitatory neuron marker CaMKIIα, and the inhibitory neuron marker GAD67 costaining in the hippocampal DG region. Scale bar: 200 μm. (I) Percentage of activated excitatory and inhibitory neurons in the hippocampal DG region. Data were collected from 3 mice per group, with 6 samples per mouse. CON, control group; MS, maternal stress group; SI, social isolation group; MS+SI, maternal stress + social isolation group; CaMKIIα, calcium/calmodulin-dependent protein kinase II α; GAD67, glutamic acid decarboxylase 67 kDa. Quantitative results are presented as mean ± SEM. Statistical significance: *P < 0.05; **P < 0.01; ***P < 0.001; ns indicates no significant difference. Statistical comparisons were made using one-way ANOVA followed by Tukey’s post hoc analysis.

Given the specific vulnerability of male offspring, we next sought to identify the neural substrate within the hippocampus underlying this effect. We first performed Nissl staining to assess neuronal structural alterations, which revealed no significant neuronal loss in hippocampal subregions (CA1, CA3, and DG) across groups (Fig. S2A to D), indicating that neither maternal stress nor adolescent social isolation induced overt cell loss. We next examined neuronal activity using c-Fos immunofluorescence staining. In the DG region, MS mice displayed a marked reduction in c-Fos^+^ cells relative to the CON mice, and this reduction was further exacerbated in MS+SI mice compared with either MS or SI mice (Fig. 2A and B). By contrast, c-Fos^+^ cell counts in CA1 and CA3 remained unchanged (Fig. S3A to D), suggesting that the DG is particularly sensitive to stress exposure. These results indicate that maternal stress selectively reduces neuronal activity in the DG, and this effect is exacerbated by subsequent adolescent social isolation. Because reduced neuronal activity could result from functional impairment or apoptosis, we next performed immunofluorescence staining using NeuN (a neuronal marker) and cleaved caspase-3 (an apoptotic marker) in the hippocampal subregions (CA1, CA3, and DG). No significant differences in cleaved caspase-3^+^/NeuN^+^ cells were observed across the groups in any of these subregions (Fig. S2E to J). Western blot analysis further confirmed no changes in the expression levels of Bax, Bcl-2, or caspase-3 among the experimental groups (Fig. S2K to M). Overall, these observations indicate that the decline in DG activity arose from functional impairments rather than neuronal apoptosis. To further assess neuronal function, we performed multielectrode array (MEA) recordings in the DG region. Heatmaps of mean firing rate and raster plots demonstrated that the mean firing rate of DG neurons was significantly decreased in MS mice relative to that in CON mice and was further reduced in MS+SI mice compared with that in either MS or SI mice. Similarly, the proportion of active area within the DG was markedly reduced in MS mice relative to that in CON mice, and these reductions were further exacerbated in MS+SI mice compared with MS or SI mice (Fig. 2C to G). These results demonstrate that maternal stress substantially diminishes neuronal excitability in the DG and that subsequent adolescent social isolation further exacerbates this impairment. Finally, to identify neuronal subtypes involved, we performed triple immunofluorescence for c-Fos, CaMKIIα (calcium/calmodulin-dependent protein kinase II α, excitatory neuronal marker) and GAD67 (glutamic acid decarboxylase 67 kDa, inhibitory neuronal marker). The analysis revealed that the vast majority of c-Fos-positive cells colocalized with CaMKIIα, whereas GAD67^+^ inhibitory neurons exhibited relatively few c-Fos signals in the DG (Fig. 2H and I). Our results indicate that excitatory neurons are the predominant contributors to DG activity and that maternal stress promotes a reduction in excitatory neuronal activation in the DG following adolescent stress in male offspring.

### Impaired synaptic plasticity increases vulnerability to adolescent stress in offspring exposed to maternal stress

**Fig. 3.**
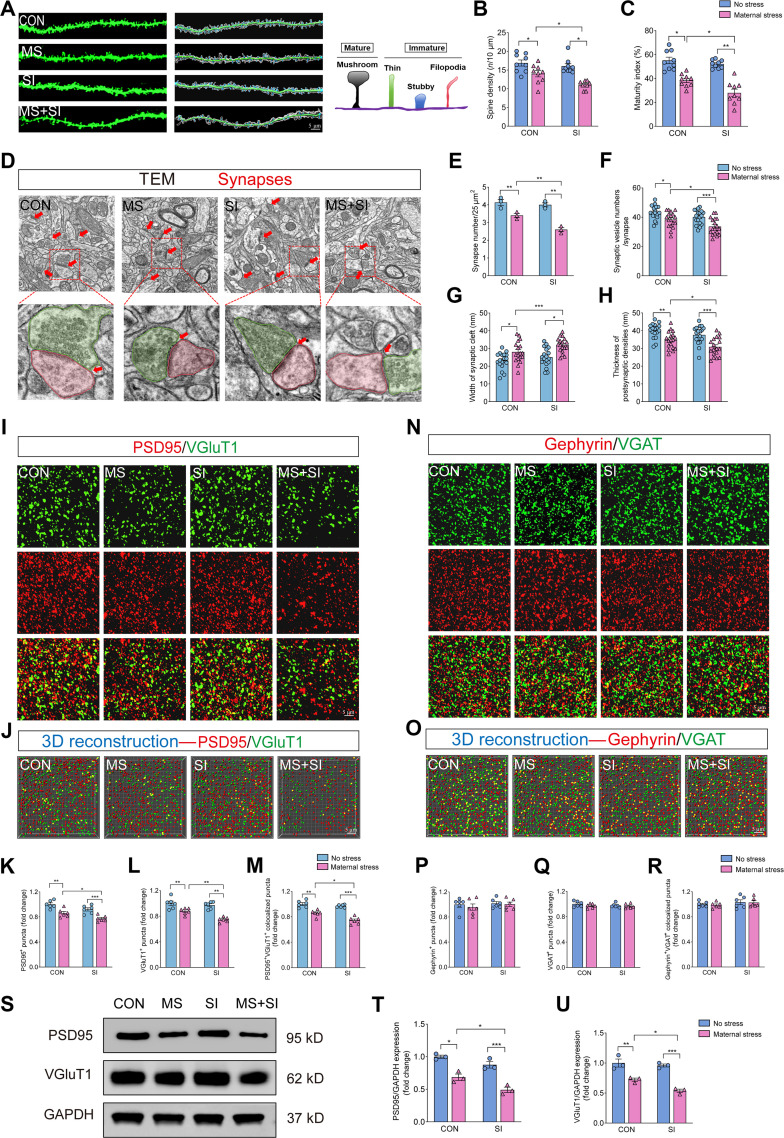
Impaired synaptic plasticity increases vulnerability to adolescent stress in offspring exposed to maternal stress. (A) Representative image of DiI staining. Scale bar: 5 μm. (B) Dendritic spine density in the hippocampal dentate gyrus (DG) region. Data were collected from 3 mice per group, with 6 samples per mouse. (C) Maturity index in the hippocampal DG region. Data were collected from 3 mice per group, with 6 samples per mouse. (D) Representative transmission electron microscopy (TEM) image of synapses in the hippocampal DG region. Scale bar: 500 nm. (E) The number of synapses in the hippocampal DG region. Data were collected from 3 mice per group, with 6 samples per mouse. (F) The count of synaptic vesicles in the hippocampal DG region. Data were collected from 3 mice per group, with 6 samples per mouse. (G) The width of the synaptic cleft in the hippocampal DG region. Data were collected from 3 mice per group, with 6 samples per mouse. (H) The thickness of postsynaptic densities in the hippocampal DG region. Data were collected from 3 mice per group, with 6 samples per mouse. (I) Representative immunofluorescence image of the excitatory postsynaptic marker PSD95 and the excitatory presynaptic marker VGluT1 in the hippocampal DG region. Scale bar: 5 μm. (J) 3-dimensional (3D) reconstruction image of the excitatory postsynaptic marker PSD95 and the excitatory presynaptic marker VGluT1 in the hippocampal DG region. Scale bar: 5 μm. (K) The number of excitatory postsynaptic PSD95^+^ puncta. (L) The number of excitatory presynaptic VGluT1^+^ puncta. (M) Quantification of PSD95^+^VGluT1^+^ colocalized puncta in the hippocampal DG region. Data show fold change relative to the control group. Data were collected from 5 mice per group, with 3 brain slices per mouse. (N) Representative immunofluorescence image of the inhibitory postsynaptic marker gephyrin and the inhibitory presynaptic marker VGAT in the hippocampal DG region. Scale bar: 5 μm. (O) 3D reconstruction of the inhibitory postsynaptic marker gephyrin and the inhibitory presynaptic marker VGAT. Scale bar: 5 μm. (P) The number of inhibitory postsynaptic gephyrin^+^ puncta. (Q) The number of inhibitory presynaptic VGAT^+^ puncta. (R) Quantification of gephyrin^+^VGAT^+^ colocalized puncta in the hippocampal DG region. Data show fold change relative to the control group. Data were collected from 5 mice per group, with 3 brain slices per mouse. (S) Representative western blot image of PSD95 and VGluT1 protein expression. (T) The protein expression levels of PSD95. (U) The protein expression levels of VGluT1. CON, control group; MS, maternal stress group; SI, social isolation; MS+SI, maternal stress + social isolation group; DiI, 1,1′-dioctadecyl-3,3,3′,3′-tetramethylindocarbocyanine perchlorate; PSD95, postsynaptic density protein 95; VGluT1, vesicular glutamate transporter 1; VGAT, vesicular GABA transporter. Quantitative results are presented as mean ± SEM. Statistical significance: *P < 0.05; **P < 0.01; ***P < 0.001; ns indicates no significant difference. Statistical comparisons were made using one-way ANOVA followed by Tukey’s post hoc analysis.

Given the neuronal excitability deficits in the DG region, we next asked whether synaptic alterations contribute to the observed vulnerability. To analyze the morphological features of dendrites and dendritic spines, we performed DiI (1,1′-dioctadecyl-3,3,3′,3′-tetramethylindocarbocyanine perchlorate) staining to assess spine density and maturity. Spine density was significantly reduced in MS mice compared with that in CON mice and was further decreased in MS+SI mice relative to that in either MS or SI alone. Dendritic spines can be classified based on their morphology into mature spines (mushroom-shaped) and immature (including thin-, stubby-, and filopodium-shaped) spines. Analysis of the maturity index revealed a significant reduction in MS mice compared with CON mice, with a further reduction observed in the MS+SI mice relative to that in either MS or SI alone (Fig. 3A to C). We next investigated whether these structural changes were accompanied by ultrastructural alterations at synapses. Transmission electron microscopy (TEM) showed that, compared to CON mice, MS mice exhibited reductions in synapse number, synaptic vesicle count, and postsynaptic density thickness, together with an increased synaptic cleft width. These changes were further exacerbated in MS+SI mice compared with those in either MS or SI alone (Fig. 3D to H). These results indicate that maternal stress induces dendritic spine loss and synaptic ultrastructural damage, which are further aggravated by adolescent social isolation.

To ascertain whether specific synaptic types were affected, we analyzed the expression of excitatory (PSD95 [postsynaptic density protein 95]/VGluT1 [vesicular glutamate transporter 1]) and inhibitory (gephyrin/VGAT [vesicular GABA transporter]) synaptic markers using immunofluorescence and 3-dimensional (3D) reconstruction via the Imaris software. Immunofluorescence staining and 3D reconstruction revealed that, compared to CON mice, MS mice exhibited a significant reduction in the number of excitatory postsynaptic markers (PSD95) and presynaptic markers (VGluT1), as well as in their colocalization, all of which were further decreased in MS+SI mice (Fig. 3I to M). Western blotting confirmed these findings, showing lower protein levels of PSD95 and VGluT1 in MS mice compared with those in CON mice, with further reductions in MS+SI mice (Fig. 3S to U). In contrast, for the inhibitory postsynaptic marker gephyrin and presynaptic marker VGAT, no significant differences were observed among the 4 groups through immunofluorescence staining and 3D reconstruction (Fig. 3N to R). These results demonstrate that maternal stress leads to a selective loss of excitatory, but not inhibitory, synapses in the DG following adolescent stress in male offspring.

### Microglial priming promotes the release of inflammatory factors and excessive phagocytosis of excitatory synapses

**Fig. 4.**
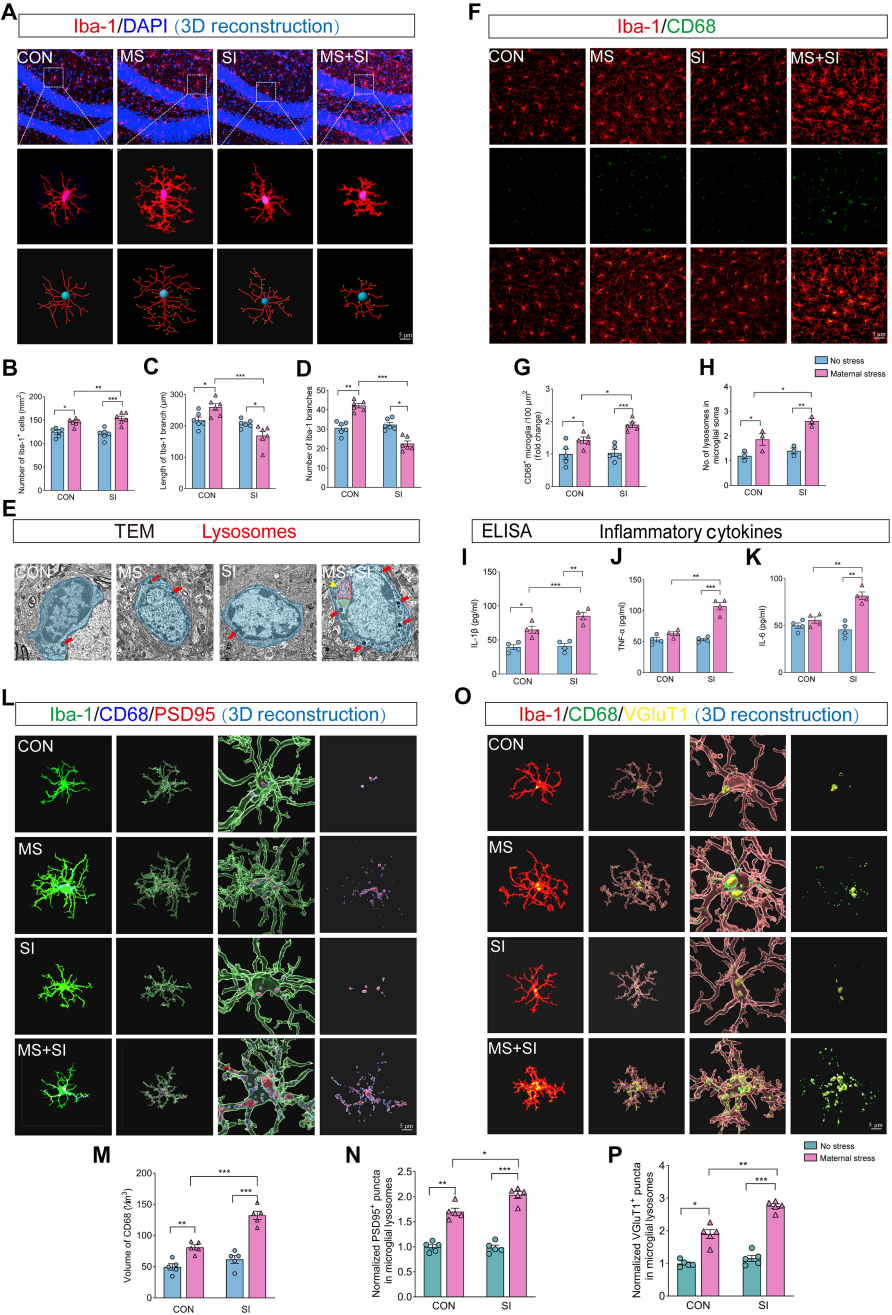
Microglial priming promotes the release of inflammatory factors and excessive phagocytosis of excitatory synapses. (A) Representative immunofluorescence image of microglial marker Iba-1 in the hippocampal dentate gyrus (DG) region. Scale bar: 100 μm. (B) The number of microglia in the hippocampal DG region. (C) The length of the microglial cell branch in the hippocampal DG region. (D) The number of microglial cell branch points in the hippocampal DG region. Data were collected from 5 mice per group, with 3 brain slices per mouse. (E) Representative transmission electron microscopy (TEM) image of lysosomes in microglial cells in the hippocampal DG region. Scale bar: 1 μm. Data were collected from 5 mice per group, with 3 brain slices per mouse. (F) Representative immunofluorescence images of microglial marker Iba-1 and lysosomal marker CD68 in the hippocampal DG region. Scale bar: 5 μm. Data were collected from 3 mice per group, with 3 brain slices per mouse. (G) The number of CD68^+^ microglial cells. Data show fold change relative to the control group. (H) The number of lysosomes in the microglial soma observed by TEM. (I) Expression of the inflammatory factor IL-1β in the hippocampal DG region. (J) Expression of the inflammatory factor TNF-α in the hippocampal DG region. (K) Expression of the inflammatory factor IL-6 in the hippocampal DG region. Data were collected from 4 mice per group, with 3 brain slices per mouse. (L) Representative immunofluorescence image of the microglial marker Iba-1, lysosomal marker CD68, and excitatory postsynaptic marker PSD95 costaining in the hippocampal DG region. Scale bar: 5 μm. (M) The volume of CD68^+^ lysosomes within microglial cells. (N) The number of PSD95^+^ excitatory postsynaptic puncta within CD68^+^ lysosomes in the microglial cells. (O) Representative immunofluorescence images of the microglial marker Iba-1, lysosomal marker CD68, and excitatory presynaptic marker VGluT1 costaining. Scale bar: 5 μm. (P) The number of VGluT1^+^ excitatory presynaptic puncta within CD68^+^ lysosomes in the microglial cells. Data were collected from 5 mice per group, with 3 brain slices per mouse. CON, control group; MS, maternal stress group; SI, social isolation group; MS+SI, maternal stress + social isolation group; PSD95, postsynaptic density protein 95; VGluT1, vesicular glutamate transporter 1; Iba-1, ionized calcium-binding adapter molecule 1. Quantitative results are presented as mean ± SEM. Statistical significance: *P < 0.05; **P < 0.01; ***P < 0.001; ns indicates no significant difference. Statistical comparisons were made using one-way ANOVA followed by Tukey’s post hoc analysis.

During brain development, synaptic pruning is critically regulated by microglia, thereby optimizing neural circuits and modulating network function. Given their central role in synaptic remodeling, we examined whether maternal stress and adolescent social isolation altered microglial state in the hippocampal DG. Immunofluorescence staining revealed an increased number of Iba-1^+^ microglia in MS mice compared with that in CON mice, accompanied by longer processes and a greater number of branches, indicative of a primed, hyper-ramified state. By contrast, microglia in MS+SI mice displayed markedly shorter processes and fewer branches than those in either MS or SI mice, consistent with a transition toward a highly activated phenotype (Fig. 4A to D). Then, to further assess microglial activation, we examined CD68, a lysosomal marker associated with phagocytic activity. Immunofluorescence staining demonstrated a significant increase in the number of CD68^+^ microglia in MS mice compared to that in CON mice, and the number was further increased in MS+SI mice compared with that in either MS or SI mice (Fig. 4F and G). To explore microglial ultrastructure, we employed TEM, which revealed a significant increase in lysosomal content in microglia from MS mice compared to that of CON mice; MS+SI mice showed even more lysosomes within their microglia compared to either MS or SI mice (Fig. 4E and H).

To determine whether these morphological changes were accompanied by inflammatory activation, we measured the levels of key pro-inflammatory cytokines using enzyme-linked immunosorbent assay (ELISA). The results indicated that IL-1β levels were significantly elevated in MS mice compared to those in CON mice, whereas IL-6 and TNF-α levels were unchanged. Additionally, IL-1β, IL-6, and TNF-α levels were markedly higher in MS+SI mice relative to those in either MS or SI mice (Fig. 4I to K). These results suggest that maternal stress primes microglia toward a pro-inflammatory state, which is further amplified by adolescent social isolation. Under stress conditions, activated microglia not only release inflammatory cytokines but also enhance their phagocytic activity. To explore the role of microglia in synaptic phagocytosis, we examined the engulfment of excitatory synapses (PSD95 and VGluT1) by microglial lysosomes. Immunofluorescence analysis with 3D reconstruction demonstrated a marked increase in excitatory synaptic components (PSD95^+^/VGluT1^+^) within microglial lysosomes in MS mice compared with CON mice, with a further increase in MS+SI mice relative to either MS or SI mice (Fig. 4L to P). By contrast, no significant differences were observed in the phagocytosis of inhibitory synapses (gephyrin^+^/VGAT^+^) among the groups (Fig. S4A to D). In summary, maternal stress primes microglia in the DG, promoting morphological activation, enhanced pro-inflammatory cytokine release, and excessive engulfment of excitatory synapses. These alterations sensitize microglia to subsequent adolescent stress and may contribute to the increased vulnerability of male offspring.

### The complement C3–C3aR pathway mediates increased stress sensitivity in adolescent male offspring following maternal stress

**Fig. 5.**
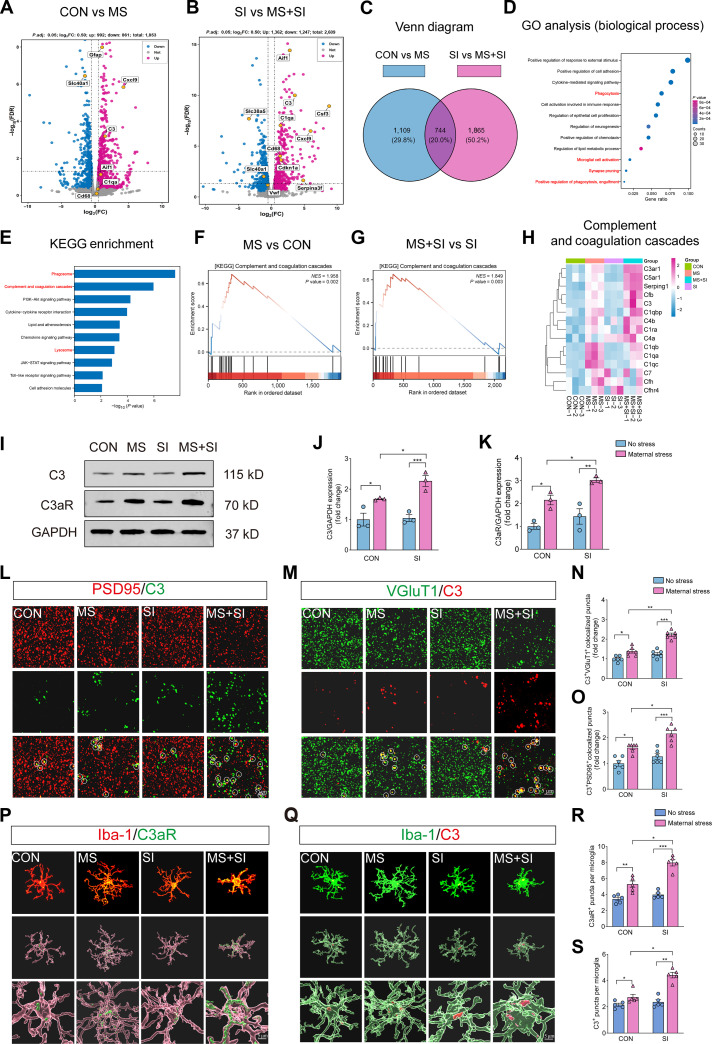
The complement C3–C3aR pathway mediates increased stress sensitivity in adolescent male offspring following maternal stress. (A) Volcano plot of differentially expressed genes in the maternal stress (MS) group vs the control (CON) group. (B) Volcano plot of differentially expressed genes in the maternal stress + social isolation (MS+SI) group vs the social isolation (SI) group. (C) Venn diagram of differentially expressed genes in the MS group vs the CON group and the MS+SI group vs the SI group. (D) Gene Ontology (GO) enrichment analysis plot of differentially expressed genes. (E) Kyoto Encyclopedia of Genes and Genomes (KEGG) pathway enrichment analysis plot of differentially expressed genes. (F) Gene set enrichment analysis (GSEA) of the complement cascade pathway in the MS group vs the CON group. (G) GSEA of the complement cascade pathway in the MS+SI group vs the SI group. (H) Heatmap of genes related to complement and coagulation cascades. Each group consists of 3 mice. (I) Representative western blot image of C3 and C3aR protein in the hippocampal dentate gyrus (DG) region. (J) The expression of C3/glyceraldehyde-3-phosphate dehydrogenase (GAPDH) in the hippocampal DG region. (K) The expression of C3aR/GAPDH in the hippocampal DG region. Each group consists of 3 mice, and 3 samples per mouse are analyzed. (L) Representative immunofluorescence images of the costaining of the excitatory postsynaptic membrane marker PSD95 and complement C3. Scale bar: 5 μm. (M) Representative immunofluorescence images of the costaining of the excitatory presynaptic marker VGluT1 and complement C3. Scale bar: 5 μm. (N) The number of C3^+^VGluT1^+^ colocalized puncta. (O) The number of C3^+^PSD95^+^ colocalized puncta. Each group consists of 6 mice, and 3 brain slices per mouse are analyzed. Data show fold change relative to the control group. (P) Representative immunofluorescence images of the costaining of the microglial marker Iba-1 and complement receptor C3aR. Scale bar: 5 μm. (Q) Representative immunofluorescence images of the costaining of the microglial marker Iba-1 and complement C3. Scale bar: 5 μm. (R) The number of C3aR^+^ puncta per microglia. (S) The number of C3^+^ puncta per microglia. CON, control group; MS, maternal stress group; SI, social isolation group; MS+SI, maternal stress + social isolation group; PSD95, postsynaptic density protein 95; VGluT1, vesicular glutamate transporter 1; Iba-1, ionized calcium-binding adapter molecule 1. Quantitative results are presented as mean ± SEM. Statistical significance: *P < 0.05; **P < 0.01; ***P < 0.001; ns indicates no significant difference. Statistical comparisons were made using one-way ANOVA followed by Tukey’s post hoc analysis.

To explore the mechanisms underlying the heightened stress sensitivity in male offspring exposed to maternal stress, we performed bulk RNA sequencing (RNA-seq) on hippocampal tissue. Volcano plot analysis identified 1,853 differentially expressed genes (DEGs) between MS and CON mice (992 up-regulated and 861 down-regulated; Fig. 5A) and 2,609 DEGs between SI and MS+SI mice (1,362 up-regulated and 1,247 down-regulated; Fig. 5B). Venn analysis revealed 744 overlapping DEGs between the 2 comparisons (Fig. 5C). Gene Ontology (GO) enrichment indicated that these DEGs were highly enriched in immune-related biological processes, including phagocytosis, microglial cell activation, synapse pruning, and positive regulation of phagocytosis and engulfment. Kyoto Encyclopedia of Genes and Genomes (KEGG) analysis further revealed significant enrichment in pathways including phagosome, lysosome, complement and coagulation cascades, and PI3K–Akt signaling (Fig. 5D and E). Notably, complement and coagulation cascades emerged as a key pathway, a finding further supported by gene set enrichment analysis, which showed significant enrichment of this pathway in MS mice relative to CON mice and in MS+SI mice relative to SI mice (Fig. 5F and G). Heatmap analysis of complement-related genes revealed robust up-regulation of several complement factors, most prominently C3 and its downstream receptor C3ar1, in MS+SI mice (Fig. 5H). Western blot analysis confirmed elevated protein levels of C3 and C3aR in MS mice relative to those in CON mice, with further increases in MS+SI mice compared with those in either MS or SI mice (Fig. 5I to K). Together, these results suggest that activation of the C3–C3aR signaling pathway mediates the heightened sensitivity to adolescent stress in male offspring following maternal stress. To investigate whether complement signaling was linked to synapse engulfment, we examined the spatial association of C3 with excitatory synapses using immunofluorescence. Compared with CON mice, MS offspring exhibited increased colocalization of complement C3 and excitatory synapses (PSD95 and VGluT1), which was further elevated in MS+SI mice (Fig. 5L to O). Moreover, immunofluorescence and 3D reconstruction revealed an increased number of C3aR^+^ and C3^+^ puncta per microglial cell in MS mice relative to that in CON mice, with further increases in MS+SI mice compared to those in either MS or SI mice (Fig. 5P to S). These findings indicate that the activation of the C3–C3aR pathway facilitates the microglial phagocytosis of excitatory synapses, thereby contributing to the increased stress vulnerability observed in adolescent offspring exposed to maternal stress.

### Pharmacological blockade of C3aR alleviates stress susceptibility in adolescent offspring following maternal stress

**Fig. 6.**
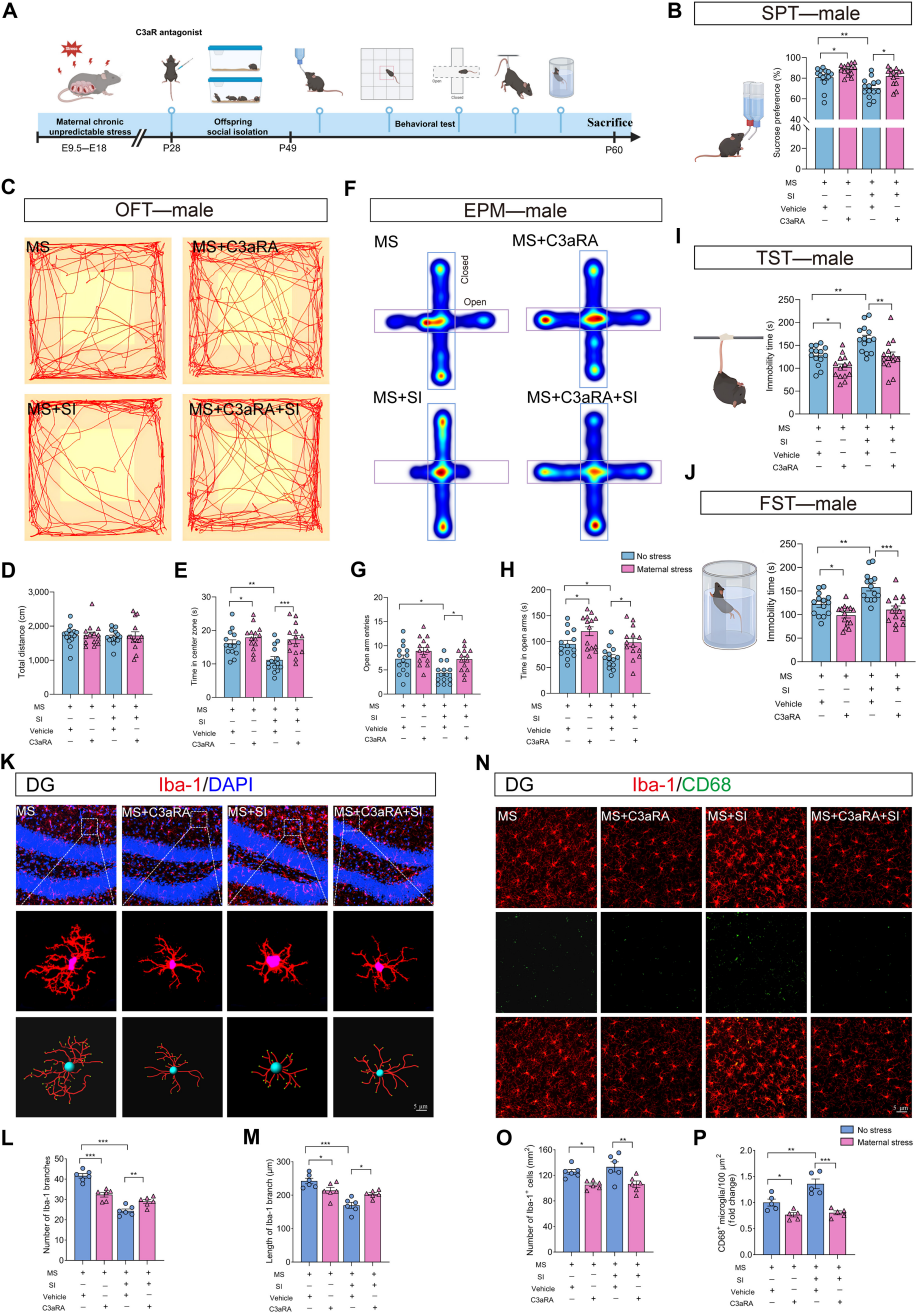
Pharmacological blockade of C3aR alleviates stress susceptibility and microglial activation in adolescent male offspring following maternal stress. (A) Schematic diagram of experimental paradigms and behavioral tests. (B) The sucrose preference ratio in the sucrose preference test (*n* = 14). (C) Representative traces of the open-field test. (D) The total distance in the open-field test (*n* = 12). (E) The time spent in the central zone in the open-field test (*n* = 12). (F) Representative traces of the elevated plus maze. (G) The number of entries into the open arms in the elevated plus maze test (*n* = 14). (H) The time spent in the open arms in the elevated plus maze test (*n* = 14). (I) The immobility time in the tail suspension test (*n* = 14). (J) The immobility time in the forced swim test (*n* = 14). (K) Representative immunofluorescence images of the microglial marker Iba-1 in the hippocampal dentate gyrus (DG) region. Scale bar: 100 μm. (L) The number of microglial cell branch points in the hippocampal DG region. (M) The length of the microglial cell branch in the hippocampal DG region. (N) Representative immunofluorescence images of the microglial marker Iba-1 and lysosomal marker CD68 in the hippocampal DG region. Scale bar: 5 μm. (O) The number of microglia in the hippocampal DG region. (P) The number of CD68^+^ microglial cells. Data show fold change relative to the control group. MS, maternal stress group; MS+SI, maternal stress + social isolation group; C3aRA, C3aR antagonist; Iba-1, ionized calcium-binding adapter molecule 1. Quantitative results are presented as mean ± SEM. Statistical significance: *P < 0.05; **P < 0.01; ***P < 0.001; ns indicates no significant difference. Statistical comparisons were made using one-way ANOVA followed by Tukey’s post hoc analysis.

**Fig. 7.**
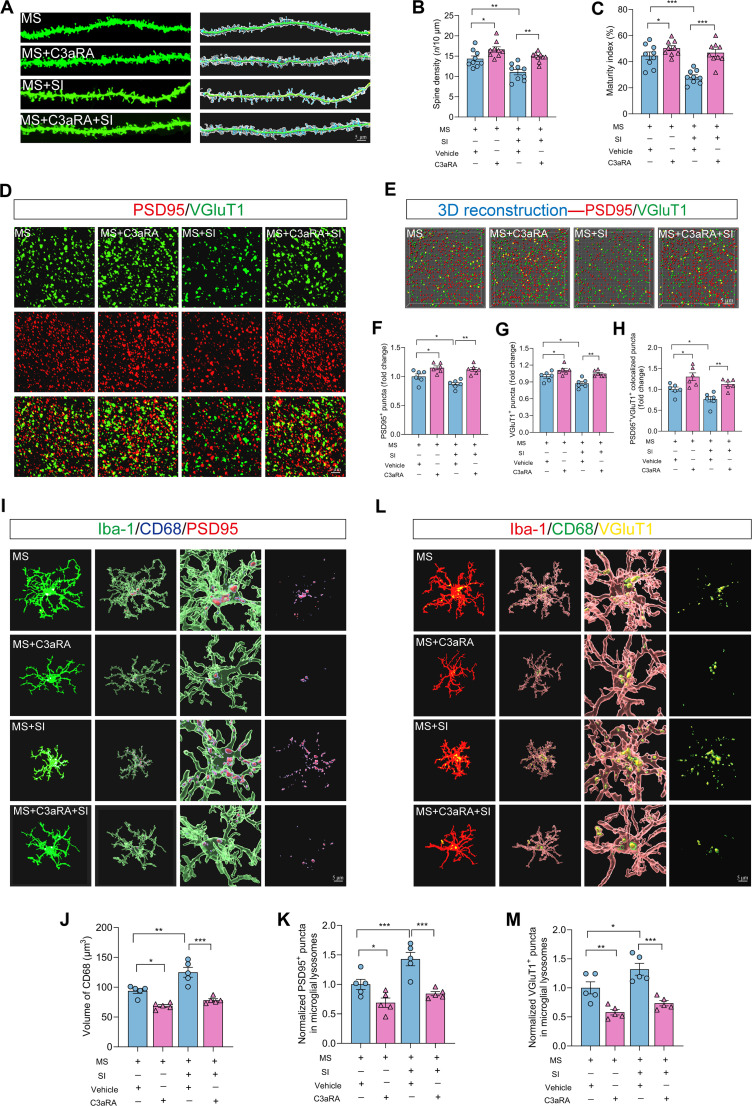
Pharmacological blockade of C3aR alleviates dendritic spine impairment and microglia-mediated loss of excitatory synapses in adolescent male offspring following maternal stress. (A) Representative image of DiI staining. Scale bar: 5 μm. (B) Dendritic spine density in the hippocampal dentate gyrus (DG) region. Data were collected from 3 mice per group, with 6 samples per mouse. (C) Maturity index in the hippocampal DG region. Data were collected from 3 mice per group, with 6 samples per mouse. (D) Representative immunofluorescence images of the excitatory postsynaptic marker PSD95 and the excitatory presynaptic marker VGluT1 in the hippocampal DG region. Scale bar: 5 μm. (E) 3-dimensional (3D) reconstruction image of the excitatory postsynaptic marker PSD95 and the excitatory presynaptic marker VGluT1 in the hippocampal DG region. Scale bar: 5 μm. (F) The number of excitatory postsynaptic PSD95^+^ puncta. (G) The number of excitatory presynaptic VGluT1^+^ puncta. (H) Quantification of PSD95^+^VGluT1^+^ colocalized puncta in the hippocampal DG region. Data show fold change relative to the control group. Data were collected from 5 mice per group, with 3 brain slices per mouse. (I) Representative immunofluorescence images of the microglial marker Iba-1, lysosomal marker CD68, and excitatory postsynaptic marker PSD95 costaining in the hippocampal DG region. Scale bar: 5 μm. (J) The volume of CD68^+^ lysosomes within microglial cells. (K) The number of PSD95^+^ excitatory postsynaptic puncta within CD68^+^ lysosomes in the microglial cells. (L) Representative immunofluorescence images of the microglial marker Iba-1, lysosomal marker CD68, and excitatory presynaptic marker VGluT1 costaining. Scale bar: 5 μm. (M) The number of VGluT1^+^ excitatory presynaptic puncta within CD68^+^ lysosomes in the microglial cells. MS, maternal stress group; MS+SI, maternal stress + social isolation group; DiI, 1,1′-dioctadecyl-3,3,3′,3′-tetramethylindocarbocyanine perchlorate; C3aRA, C3aR antagonist; PSD95, postsynaptic density protein 95; VGluT1, vesicular glutamate transporter 1; Iba-1, ionized calcium-binding adapter molecule 1. Quantitative results are presented as mean ± SEM. Statistical significance: *P < 0.05; **P < 0.01; ***P < 0.001; ns indicates no significant difference. Statistical comparisons were made using one-way ANOVA followed by Tukey’s post hoc analysis.

Given the role of the C3–C3aR pathway in mediating stress susceptibility, we next tested whether pharmacological blockade of C3aR could reverse these effects. A C3aR antagonist (C3aRA; 1 mg/kg, intraperitoneally) was administered to offspring starting at P28, followed by 3 weeks of social isolation and behavioral testing (Fig. 6A). C3aR blockade had no effect on locomotor activity, as the total distance traveled in the OFT was comparable across groups (Fig. 6C and D). However, relative to MS+vehicle+SI mice, MS+C3aRA+SI mice spent more time in the center zone in the OFT (Fig. 6C to E), explored open arms more frequently and for longer durations in the EPM (Fig. 6F to H), showed increased sucrose preference in the SPT (Fig. 6B), and displayed reduced immobility in both the TST and FST (Fig. 6I and J). These results indicate that pharmacological blockade of C3aR alleviates anxiety- and depression-like behaviors induced by maternal stress and adolescent social isolation, thereby reducing stress susceptibility in male offspring. We next examined microglial states in the hippocampal DG. Immunofluorescence analysis revealed that, compared to those of MS+vehicle+SI mice, the microglia of MS+C3aRA+SI mice exhibited notable morphological changes, characterized by an increased number of branches and longer processes (Fig. 6K to M). Additionally, the overall number of microglia was significantly reduced in MS+C3aRA+SI mice compared to that in MS+vehicle+SI mice (Fig. 6O), together suggesting a shift toward a more homeostatic, ramified state. To further assess microglial activation, we examined CD68 expression, a lysosomal marker associated with phagocytic activity. The results showed a significant decrease in the number of CD68^+^ microglia in the hippocampal DG of MS+C3aRA+SI mice relative to that in MS+vehicle+SI mice (Fig. 6N and P). Our findings indicate that C3aR blockade suppresses microglial activation in the hippocampus DG region of male offspring exposed to maternal stress, thereby reducing their vulnerability to adolescent stress. We then investigated whether C3aR blockade influences synaptic plasticity. DiI staining revealed that MS+C3aRA+SI mice exhibited a significant increase in dendritic spine density and maturity index compared to MS+vehicle+SI mice (Fig. 7A to C). Moreover, immunofluorescence analysis showed a significant increase in the number of the excitatory postsynaptic marker PSD95 and excitatory presynaptic marker VGluT1 in MS+C3aRA+SI mice, along with enhanced colocalization, compared to that in MS+vehicle+SI mice (Fig. 7D to H). Furthermore, we assessed the microglial phagocytosis of synapses. Immunofluorescence analysis with 3D reconstruction indicated that MS+C3aRA+SI mice displayed a significant reduction in CD68^+^ lysosomal volume, together with fewer engulfed PSD-95^+^ and VGluT1^+^ puncta within microglial lysosomes, compared with MS+vehicle+SI mice (Fig. 7I to M). These findings suggest that blockade of C3aR reduces the phagocytic activity of microglia in the hippocampal DG, which in turn mitigates the synaptic loss and reduces susceptibility to adolescent stress in male offspring exposed to maternal stress.

### Activation of hippocampal DG glutamatergic neurons reduces stress susceptibility in adolescent offspring following maternal stress

**Fig. 8.**
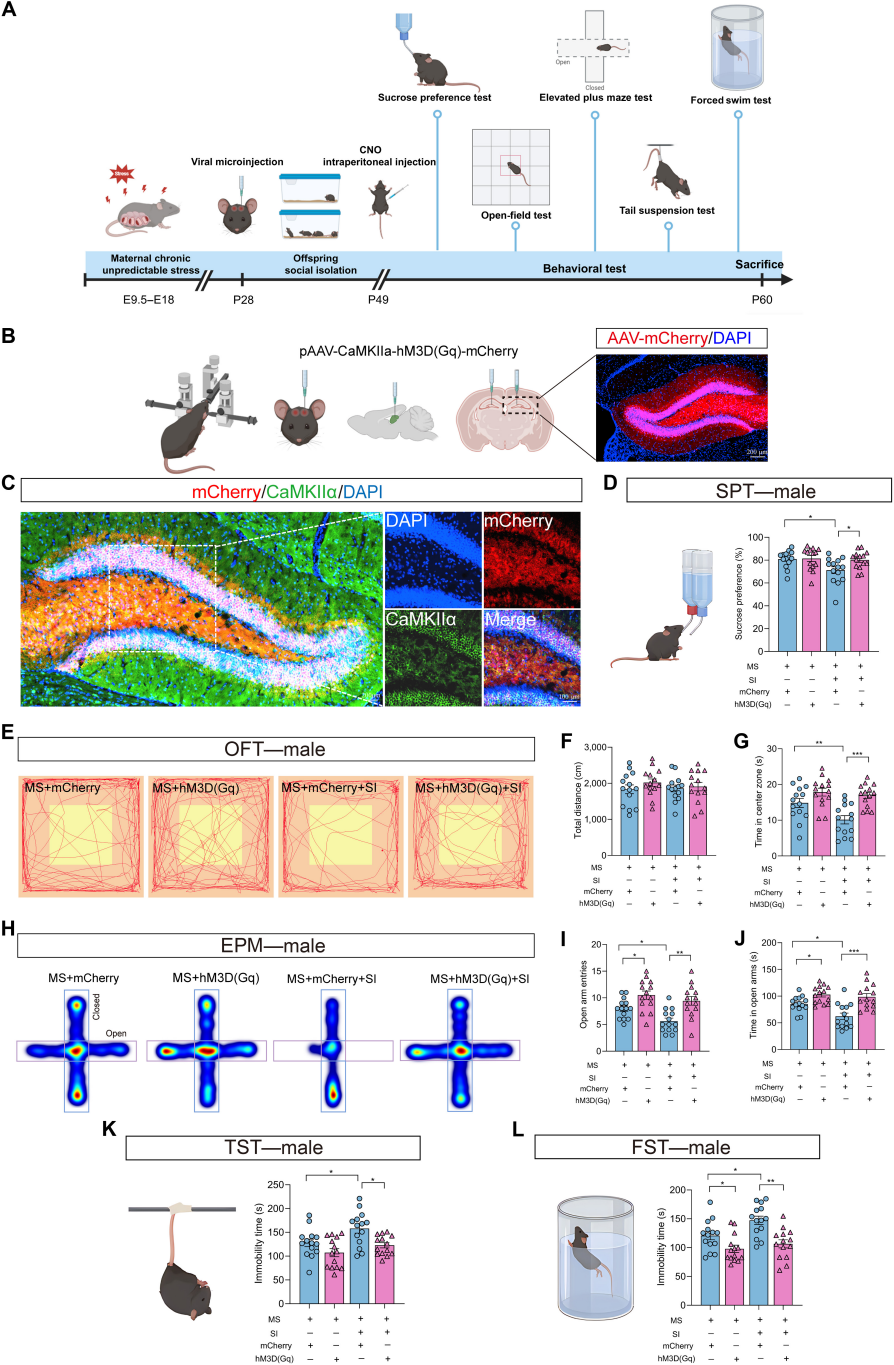
Activation of hippocampal dentate gyrus (DG) glutamatergic neurons reduces stress susceptibility in adolescent male offspring following maternal stress. (A) Schematic diagram of experimental paradigms and behavioral tests. (B) Injection site and viral expression. Scale bar: 200 μm. (C) Representative immunofluorescence images showing the colocalization of virus-expressed hM3D(Gq)-mCherry and the excitatory neuron marker CaMKIIα in the hippocampal DG region. Scale bar: 100 μm. (D) The sucrose preference ratio in the sucrose preference test (*n* = 14). (E) Representative traces of the open-field test. (F) The total distance in the open-field test (*n* = 14). (G) The time spent in the central zone in the open-field test (*n* = 14). (H) Representative traces of the elevated plus maze. (I) The number of entries into the open arms in the elevated plus maze test (*n* = 14). (J) The time spent in the open arms in the elevated plus maze test (*n* = 14). (K) The immobility time in the tail suspension test (*n* = 14). (L) The immobility time in the forced swim test (*n* = 14). MS, maternal stress group; SI, social isolation group. Quantitative results are presented as mean ± SEM. Statistical significance: *P < 0.05; **P < 0.01; ***P < 0.001; ns indicates no significant difference. Statistical comparisons were made using one-way ANOVA followed by Tukey’s post hoc analysis.

Given the observed reduction in glutamatergic signaling in the hippocampal DG of MS offspring, we next explored whether enhancing the activity of DG excitatory neurons could alleviate their susceptibility to adolescent stress (Fig. 8A). We performed viral microinjections of AAV-CaMKIIα-hM3D(Gq)-mCherry into the hippocampal DG, enabling chemogenetic activation of excitatory neurons upon ligand binding. Successful viral transduction was confirmed by robust mCherry expression restricted to the hippocampus DG (Fig. 8B). To verify neuronal targeting, we examined the colocalization of mCherry with CaMKIIα, a marker of excitatory neurons, and observed strong overlap, confirming selective targeting of DG excitatory neurons (Fig. 8C). We next conducted a series of behavioral tests to assess anxiety- and depression-like behaviors. Locomotor activity was unaffected, as no group differences were observed in total distance traveled in the OFT, confirming that chemogenetic activation did not induce motor abnormalities or pathological hyperexcitability. However, compared with MS+mCherry+SI mice, MS+hM3D(Gq)+SI mice spent significantly more time in the center zone of the OFT (Fig. 8E to G) and also exhibited increased open-arm exploration in the EPM, as indicated by both longer durations and more entries (Fig. 8H to J). In the SPT, MS+hM3D(Gq)+SI mice showed a significantly higher preference for sucrose than MS+mCherry+SI mice (Fig. 8D). Likewise, in both the TST and FST, immobility times were markedly shorter in MS+hM3D(Gq)+SI mice compared with those in MS+mCherry+SI mice (Fig. 8K and L). Collectively, these behavioral results demonstrate that chemogenetic activation of glutamatergic neurons in the hippocampal DG significantly ameliorates “2-hit” stress-induced anxiety- and depression-like behaviors, highlighting the critical role of hippocampal excitatory neurons in modulating stress sensitivity and emotional regulation following maternal stress.

## Discussion

**Fig. 9.**
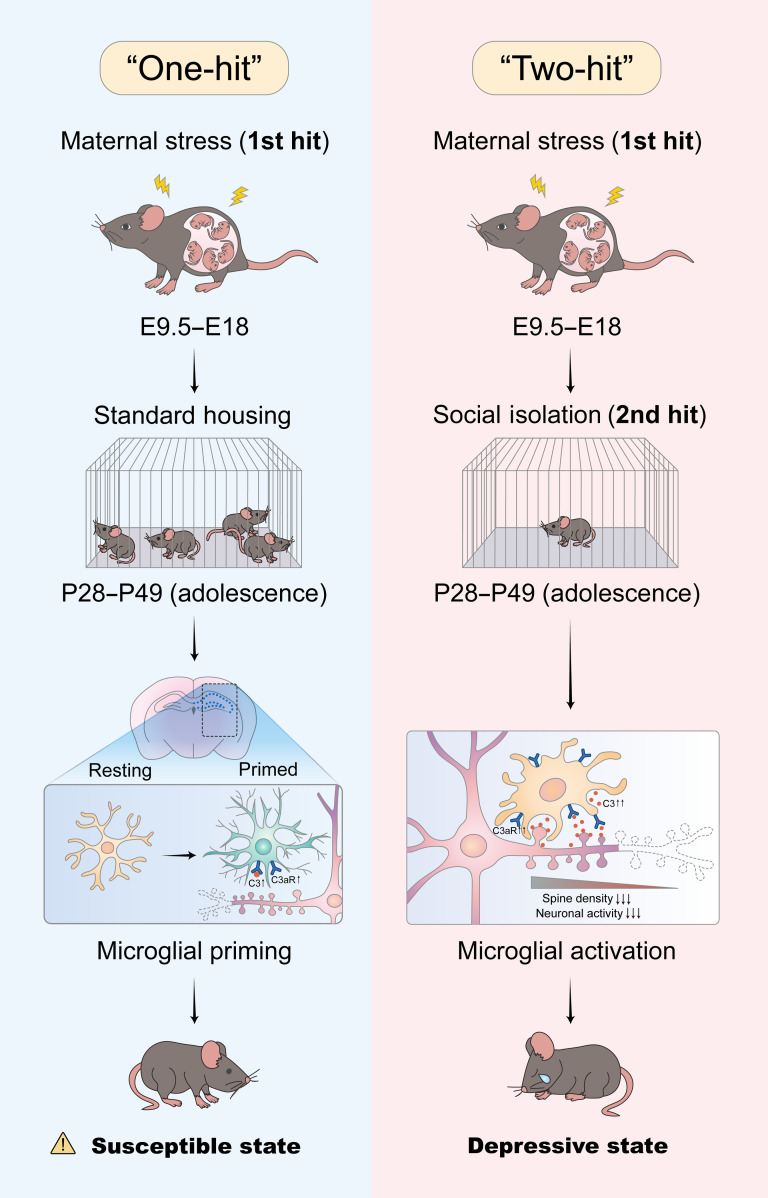
Maternal chronic unpredictable stress during gestation (embryonic days 9.5 to 18) primes microglia in the offspring hippocampal dentate gyrus. Following adolescent social isolation (postnatal days 28 to 49), primed microglia drive complement C3–C3aR-dependent phagocytosis of excitatory synapses and reduce glutamatergic neuronal activity in the dentate gyrus, producing anxiety- and depressive-like behaviors in male—but not female—offspring. Pharmacological blockade of C3aR or chemogenetic activation of dentate gyrus glutamatergic neurons during adolescence alleviates this stress susceptibility.

Our study demonstrates that maternal stress selectively enhances stress susceptibility in male adolescent offspring through complement C3–C3aR-mediated microglial priming in the hippocampal DG region. This prenatal programming establishes a latent vulnerability state that, upon subsequent adolescent social isolation, drives excessive microglial phagocytosis of excitatory synapses, reduces glutamatergic neuronal excitability, and precipitates depression-like behaviors. Critically, we show that this pathological cascade is reversible: either pharmacological C3aR antagonism or chemogenetic activation of DG glutamatergic neurons during adolescence rescues stress-induced deficits. These findings establish complement-mediated synaptic pruning as a central mechanism linking prenatal adversity to adolescent psychopathology and identify the adolescent period as a therapeutic window for preventing stress-related mood disorders in mice (Fig. 9).

The rising prevalence of adolescent depression underscores the urgent need to understand how early-life adversity programs enduring vulnerability to stress-related disorders. The “2-hit” hypothesis provides a mechanistic framework, positing that initial adverse experiences sensitize neural systems to subsequent environmental challenges [36–39]. Here, we employed a developmentally informed “2-hit” paradigm combining chronic unpredictable maternal stress (E9.5 to E18) with adolescent social isolation (P28 to P49), targeting 2 critical windows of heightened neural plasticity. Our chronic unpredictable stress protocol more faithfully models the multifaceted psychosocial stressors encountered during human pregnancy than single-modality paradigms such as pharmacological manipulations, capturing the complex, variable nature of real-world maternal adversity. Adolescent social isolation served as a subthreshold “second hit”—insufficient to induce pathology in control animals, but potent in unmasking vulnerability in maternally stressed offspring. Importantly, the unpredictable nature of our maternal stress protocol is critical: whereas predictable early-life stress can paradoxically confer resilience through adaptive recalibration of stress-response systems [40], unpredictable stress disrupts normal developmental programming, establishing maladaptive priming that persists into adolescence. Our findings extend this framework by demonstrating that chronic unpredictable maternal stress induces sex-specific microglial priming in male offspring, thereby amplifying their susceptibility to adolescent social stress.

A critical distinction in our findings is that maternal stress induces microglial priming rather than full activation. Microglial priming refers to a sensitized, preconditioned state in which cells exhibit a lowered activation threshold and exaggerated responses to subsequent stimuli without overt inflammatory activity [41]. In contrast, activation represents a complete effector response to immediate stimuli, characterized by dramatic morphological changes, robust cytokine release, and active phagocytosis [42]. This distinction is not merely semantic; it fundamentally shapes our understanding of how early-life adversity establishes latent vulnerability that manifests only upon subsequent challenge—the essence of the “2-hit” hypothesis. Our data provide multiple lines of evidence that maternal stress induces microglial priming rather than full activation. First, at the molecular level, MS offspring exhibited up-regulation of complement components (C3 and C3aR) and moderate increases in lysosomal markers (CD68) but did not show significant elevation of pro-inflammatory cytokines in the absence of adolescent stress. Second, morphologically, microglia in MS-only mice displayed subtle changes, including increased ramification and longer processes, yet retained a highly branched morphology and did not adopt the amoeboid phenotype characteristic of fully activated microglia. This intermediate morphological state further supports a primed rather than activated phenotype. Third, and most critically, the functional consequences align with priming: maternal stress alone did not induce significant behavioral deficits. Only when adolescent social isolation was superimposed did we observe exaggerated microglial responses—including pronounced morphological activation, excessive synaptic phagocytosis, and behavioral pathology. Importantly, microglial priming is not universally maladaptive. Controlled early-life immune challenges can establish “trained immunity”, wherein microglia acquire enhanced capacity to clear pathological proteins such as amyloid-β, conferring neuroprotection in later life. However, in the context of chronic unpredictable stress and psychiatric vulnerability, the priming state we identified appears decidedly maladaptive, predisposing circuits to excessive activity-dependent pruning during adolescent refinement [43,44]. This underscores the context-dependent nature of microglial priming: its functional consequences hinge critically on the nature, timing, and intensity of secondary challenges, as well as the developmental stage during which they are encountered.

Beyond establishing the priming nature of microglial alterations, our bulk RNA-seq analysis revealed a coordinated, multilayered neuroimmune reprogramming induced by maternal stress. The GO enrichment analysis prominently highlighted “phagocytosis”, “microglial cell activation”, and “synapse pruning”, independently corroborating our core finding of aberrant microglial engulfment of synapses at the transcriptomic level. Notably, complement pathway activation was not restricted to the C3–C3aR axis. The heatmap revealed coordinated up-regulation of the entire classical complement cascade, including the upstream initiators C1q (C1qa, C1qb, and C1qc), the regulator Serping1, and downstream components C4a, C4b, and C7, suggesting that the complete classical cascade participates in maternal-stress-induced synaptic remodeling. The concurrent up-regulation of C5ar1 further suggests potential involvement of the C5a–C5aR1 axis, opening new avenues for future investigation. KEGG analysis identified enrichment of the phagosome and lysosome pathways (Fig. 5E), consistent with our observation of increased CD68^+^ lysosomal content and excessive synaptic engulfment. The volcano plots also showed marked up-regulation of Aif1 (Iba1), Cd68, and Gfap, independently validating microglial and astrocytic reactivity. Of particular note, the enrichment of the PI3K–Akt pathway—located downstream of C3aR—may mediate signal transduction from complement receptor activation to phagocytic effector function, providing a potential molecular link between C3aR engagement and excessive synaptic pruning. We focused our mechanistic validation on the C3–C3aR axis given its most prominent up-regulation, well-established mechanistic role in microglia-dependent synaptic elimination, and the availability of a pharmacological antagonist for causal validation. Nevertheless, the other enriched pathways and genes likely contribute to the complex molecular landscape of stress susceptibility, and future studies employing single-cell RNA-seq or spatial transcriptomics will be essential to dissect cell-type-specific contributions and further refine this molecular map.

Microglia execute synaptic pruning through multiple molecularly distinct pathways that collectively orchestrate the identification, tagging, and elimination of synapses during circuit refinement [45–47]. The classical complement cascade operates via opsonization: complement proteins C1q and C3 tag synapses for elimination, which are subsequently recognized by microglial complement receptors (CR3 and C3aR) and engulfed via receptor-mediated phagocytosis [48–50]. The TREM2–phosphatidylserine axis enables microglia to detect and remove synapses exposing phosphatidylserine, a canonical “eat-me” signal on apoptotic or compromised synaptic elements; TREM2 deficiency impairs hippocampal pruning, confirming its nonredundant role in synaptic homeostasis [51,52]. The CX3CR1–fractalkine pathway governs microglial–neuronal cross talk, with CX3CR1 knockout mice exhibiting aberrant pruning and disrupted connectivity [53]. Counterbalancing these elimination signals, “don’t-eat-me” ligands such as CD47 and CD200 engage inhibitory receptors (SIRPα and CD200R) on microglia, protecting active, functional synapses from inappropriate removal [54]. Our findings implicate the C3–C3aR axis as the primary mediator of stress-induced synaptic pathology. Maternal stress selectively up-regulated C3 and C3aR in the DG without broadly activating other pruning pathways, suggesting pathway-specific priming. Upon adolescent stress, this primed complement machinery drove exaggerated engulfment of excitatory synapses, reducing dendritic spine density and glutamatergic transmission. Critically, pharmacological C3aR blockade substantially rescued synaptic and behavioral deficits, establishing causal necessity for complement signaling in mediating stress-induced pathology. The anatomical and cell-type specificity of this mechanism—restriction to hippocampal DG glutamatergic synapses in males—highlights the precision with which developmental stress sculpts circuit-level vulnerability. This cellular specificity extends to the source of complement itself: our colocalization analyses indicate that astrocytes are the predominant source of C3 in the hippocampal DG under our experimental conditions (Fig. 3R). Nevertheless, it is well established that microglia can also produce complement components, including C3, particularly under inflammatory or disease-associated states [33,55]. Thus, although astrocyte-derived C3 likely constitutes the major opsonizing signal in our model, a contribution from microglial autocrine or paracrine C3 production cannot be excluded and may further amplify local complement-mediated synaptic pruning. Cell-type-specific genetic manipulation of C3, such as conditional deletion in astrocytes versus microglia, will be needed to precisely delineate the relative contributions of these cellular sources to stress-induced synaptic pathology.

Adolescence represents a unique reversible critical period characterized by heightened neuroplasticity, conferring both vulnerability and opportunities for intervention. Whereas classical critical periods are restricted to specific sensory systems (e.g., the visual cortex), the adolescent window is defined by the protracted maturation of higher-order brain regions. Specifically, this period is critical for the development of the prefrontal cortex and hippocampus—structures governing executive function, emotional regulation, and social cognition [56–58]. During this period, the neurobiological mechanisms that support critical period plasticity—including adjustments in E/I balance, ongoing synaptic pruning, and dynamic chromatin remodeling—are highly active, rendering neural circuits exceptionally sensitive to environmental influence [59–61]. Our “2-hit” model—maternal stress followed by adolescent social isolation—demonstrates that adolescence is a critical vulnerable window for stress susceptibility. Social isolation alone induced minimal changes, confirming its role as a subthreshold stressor that only precipitates pathology in maternally primed individuals. This temporal specificity suggests that maternal stress establishes a dormant primed microglial state that is reactivated during adolescence, when active synaptic refinement renders circuits vulnerable to aberrant pruning. Critically, this pathological trajectory is reversible. Both C3aR inhibition and chemogenetic DG neuronal activation during adolescence substantially normalized behavioral deficits, microglial morphology, and synaptic density. This reversibility demonstrates that adolescent plasticity is bidirectional: while negative experiences derail development, targeted interventions delivered within this window can effectively reverse pathological trajectories, supporting resilience during the assessed adolescent period. Whether these protective effects persist into adulthood remains an important open question. Specifically, longitudinal follow-up studies extending into adulthood, combined with exposure to a subsequent “third-hit” stressor, will be essential to determine whether adolescent C3aR antagonism or chemogenetic neuronal activation confers durable resilience or whether susceptibility re-emerges once the intervention is withdrawn or upon renewed stress challenge. Resolving this question is critical for evaluating the long-term therapeutic potential of interventions delivered within the adolescent critical window.

Our findings reveal sex-dependent differences in stress sensitivity, with male offspring showing greater vulnerability than females. While epidemiological studies report higher depression rates in adolescent females, particularly in later adolescence (ages 15 to 18), our findings focus on early adolescence (PNDs 28 to 49, corresponding to human ages 12 to 17). During this earlier window, clinical studies have shown that boys may exhibit comparable or even greater vulnerability to certain stressors, particularly social stress. The sex differences we observed are likely driven by multiple mechanisms, including variations in fetal neurodevelopment, hormonal regulation, and epigenetic modifications. Previous research suggests that male fetuses develop more slowly in utero and exhibit poorer adaptation to stress compared to females, making them more susceptible to environmental challenges [62]. This slower development may render males more vulnerable to stress during both early and later stages of life. Additionally, maternal stress is thought to induce telomere shortening in male fetuses, reflecting a greater biological burden, which could contribute to their heightened stress sensitivity [63]. Sex hormones, particularly testosterone, play a crucial role in the neurodevelopment of emotion-related brain regions such as the hippocampus, amygdala, and prefrontal cortex in male fetuses [64,65]. Maternal stress may disrupt testosterone stability, leading to aberrant neurodevelopment and an increased sensitivity to stress during adolescence [66]. In contrast, estrogen in female fetuses exerts neuroprotective effects by promoting synaptic plasticity, enhancing neurogenesis, and partially inhibiting inflammatory responses. These neuroprotective actions may help mitigate the negative effects of maternal stress on female offspring [67,68]. Finally, epigenetic mechanisms also play a critical role in mediating sex differences in stress sensitivity. Studies have shown that female fetuses exhibit more pronounced epigenetic protective mechanisms, such as elevated levels of H3K27me3 modifications in the placenta, which are thought to confer greater resilience to maternal stress. These epigenetic changes may contribute to the enhanced ability of females to buffer against the effects of early-life stress. Beyond these fetal programming mechanisms, the HPA axis represents another critical mediator likely to underlie the sex-specific vulnerability observed in our model [69]. As the principal neuroendocrine system governing stress responsivity, the HPA axis exhibits pronounced sexual dimorphism in both its baseline set point and its reactivity to environmental challenge, and prenatal stress is well documented to durably reprogram its function in a sex-dependent manner. Such reprogramming could plausibly converge with the complement–microglial mechanism identified here, as glucocorticoids directly modulate microglial reactivity and complement expression, potentially lowering the threshold for maladaptive priming selectively in male offspring. Although the present study focused on the hippocampal complement–microglial axis, a comprehensive characterization of HPA axis function—including basal and stress-evoked corticosterone levels, glucocorticoid receptor expression, and central corticotropin-releasing hormone signaling—across both sexes will be essential to fully delineate the sex-specific mechanisms driving differential stress vulnerability [70,71]. Future studies should investigate the specific biological mechanisms underlying the sex differences in stress-related mood disorders among offspring exposed to prenatal stress.

Our finding that maternal stress selectively affects the DG, while sparing CA1 and CA3, aligns with the unique developmental and molecular features of this hippocampal subregion. The DG is the only hippocampal subregion that retains active neurogenesis throughout postnatal development and adolescence. This continuous integration of newborn neurons requires sustained microglial involvement in synaptic remodeling, placing DG microglia in a heightened baseline state of pruning activity compared with the more quiescent microglia in CA1 and CA3, which may render them more susceptible to stress-induced priming. This functional predisposition is further reinforced by the unique anatomical position of the DG as the gateway of the hippocampal trisynaptic circuit, where granule cells receive direct cortical input via the perforant path and bear disproportionate excitatory loads and plasticity demands, making them particularly vulnerable to E/I imbalance caused by aberrant synaptic pruning. In addition, the DG harbors a unique neurogenic microenvironment, in which the subgranular zone is enriched in trophic factors and immune signaling molecules that actively modulate microglial function. Maternal stress may reprogram this microenvironment, thereby selectively priming DG microglia. We acknowledge that these interpretations are grounded in published evidence, and the molecular mechanisms underlying DG-selective vulnerability remain to be directly verified experimentally. Comparative transcriptomic profiling of microglia isolated from distinct hippocampal subregions in future research will be critical to fully unravel this regional specificity. Beyond the mechanistic insights provided by these findings, the complement and microglial markers identified in this work hold promising translational potential as candidate biomarkers for at-risk population stratification. Circulating C3 and its cleavage products, indicators of peripheral complement activation, can be readily quantified in blood and have been linked to mood and psychotic disorders in clinical cohorts. Similarly, translocator protein ligands detectable via positron emission tomography imaging serve as accessible surrogate markers reflecting central neuroimmune activation [72]. Although the link between peripheral and central complement signaling requires validation, our findings raise the possibility that a combined panel of complement and neuroimmune markers could aid the early identification of adolescents harboring latent stress vulnerability, enabling timely intervention during this critical window. Prospective clinical studies will be required to evaluate this translational potential.

In conclusion, our study demonstrates that maternal stress primes male offspring for heightened stress vulnerability through complement C3–C3aR-mediated microglial synaptic pruning. Critically, we identify adolescence as a reversible critical window during which targeted interventions can effectively reverse these pathological changes. These findings advance our mechanistic understanding of the developmental origins of depression and suggest a potential preventive framework: identifying at-risk adolescents through biomarker screening and intervening during the plastic adolescent window with C3aR-targeted therapies. Future studies extending follow-up into adulthood will be essential to determine whether such interventions confer long-term protection against mood disorders. By bridging neurodevelopmental biology with clinical psychiatry, our work opens new avenues for tackling one of the most pressing public health challenges of our time.

## Materials and Methods

### Animals

All animal husbandry and experimental procedures were conducted in strict accordance with the guidelines of the Animal Ethics Committee at Fudan University. Mice were housed under standardized laboratory conditions with a 12-h light/dark cycle (lights on at 8:00 AM, lights off at 8:00 PM), a temperature of 23 to 25 °C, and humidity of 40% to 60%. The animals had free access to food and water. The C57BL/6J wild-type mice were purchased from Shanghai SLAC Laboratory Animal Co., Ltd. and acclimated to the environment for at least 1 week before assignment to experimental groups. After mice attained sexual maturity (6 to 8 weeks), breeding was conducted by pairing 2 females with 1 male. Vaginal plug examination was performed on the following day. Females showing positive vaginal plugs were marked as E0.5 and housed individually in isolation cages thereafter.

### “Two-hit” paradigm

To investigate the impact of maternal stress on offspring stress susceptibility, we constructed a developmental “2-hit” model involving chronic unpredictable stress during pregnancy and subsequent social isolation of adolescent offspring. The experiment included 4 groups: (a) control (CON): pregnant mice without chronic unpredictable stress, adolescent offspring housed in groups; (b) maternal stress (MS): pregnant mice with chronic unpredictable stress, offspring housed in groups; (c) social isolation (SI): pregnant mice without chronic unpredictable stress, offspring socially isolated for 3 weeks starting at P28; and (d) maternal stress + social isolation (MS+SI): pregnant mice with chronic unpredictable stress, offspring socially isolated for 3 weeks starting at P28.

### Maternal chronic unpredictable stress model

Based on previous literature, to better simulate real-life stress environments during pregnancy, we adopted the maternal chronic unpredictable stress model [73]. Pregnant mice were exposed to various stressors between embryonic days 9.5 and 18 (E9.5 to E18). The stressors included restraint stress, cage tilting, wet bedding, and nocturnal lighting, as detailed in Fig. S1A. The stressors were applied in a random order to ensure unpredictability and to prevent adaptive responses in the mice.

### Adolescent social isolation stress model

The offspring mice were weaned at 3 weeks of age. To assess the sensitivity to stress, the offspring underwent 3 weeks of social isolation stress starting at P28 as described in a previous study [20]. The experimental group was individually housed for 3 weeks, while the CON group remained group-housed (4 to 5 mice per cage). This model simulates social deprivation and isolation during adolescence.

### Behavior test

Prior to all behavioral assessments, mice were transferred to a buffer room at least 12 h in advance to allow for environmental acclimatization. During the experiments, ambient light intensity was maintained at a low level (60 lux) to minimize anxiety-related responses and to ensure the stability of the testing environment. To maintain consistency across experimental conditions, all behavioral tests were conducted during the light phase of the light–dark cycle, between 9:00 AM and 6:00 PM. All behavioral assessments were performed following the 3-week social isolation period (P28 to P49). Tests were administered in a fixed sequence proceeding from the least to the most stressful to minimize carryover effects between assessments: SPT (P50 to P53), OFT (P55), EPM (P56), TST (P58), and FST (P60) [74–76]. Each animal was tested only once per paradigm, and all apparatus were thoroughly cleaned with 75% ethanol between individual trials to eliminate olfactory cues (Fig. 1A). Moreover, throughout both the experimental procedures and subsequent data analyses, experimenters were blinded to the group assignments of the mice in order to minimize potential bias.

### Sucrose preference test

The SPT was conducted using 2 bottles: one containing a 1% sucrose solution and the other containing tap water. Prior to formal testing, mice underwent a 24-h adaptation period during which the positions of the bottles were randomly alternated to eliminate potential environmental bias. Following adaptation, a 12-h period of food and water deprivation was applied to ensure that all mice were in a comparable physiological state at the onset of testing. At the beginning of the test, the sucrose and water bottles were randomly assigned to either the left or right side of the cage, allowing unrestricted access. The test lasted for 6 h, during which the animals were not disturbed. To measure sucrose (*B*) and water (*C*) intake, each bottle was weighed before the test commenced and after its completion. The sucrose preference index (*A*) was calculated as *A* = *B*/(*B* + *C*) × 100%. Upon completion of the test, mice were returned to their standard housing conditions, and the bottles were thoroughly cleaned to prevent cross-contamination and to prepare the apparatus for subsequent trials.

### Open-field test

The experiment was conducted in a closed, opaque chamber (40 × 40 × 40 cm), with the central zone representing 25% of the total area (20 × 20 cm). At the onset of each trial, the mouse was gently positioned in the central zone, and its spontaneous locomotor activity was monitored for 5 min using the EthoVision XT 8.5 software (Noldus, Wageningen, the Netherlands). The software continuously tracked the animal’s movements and quantified both the time spent in the central zone and the total distance traveled. To eliminate potential olfactory confounds and maintain experimental consistency, all surfaces of the apparatus were thoroughly cleaned with 75% ethanol following each trial.

### Elevated plus maze

The experiment was conducted in an EPM, consisting of 2 closed arms and 2 open arms. The apparatus was raised 60 cm above the floor to create an elevated environment. Each arm measured approximately 30 cm in length and 5 cm in width, with a central platform of 5 × 5 cm. At the beginning of each trial, a mouse was placed individually on the central platform, facing one of the open arms. During testing, spontaneous locomotor activity was recorded for 5 min using the EthoVision XT 8.5 software (Noldus, Wageningen, the Netherlands), which quantified both the time spent in the open arms and the number of entries into the open arms. After each trial, the maze was cleaned with 75% ethanol to remove olfactory cues and prevent interference with subsequent tests.

### Tail suspension test

The mouse’s tail was suspended using adhesive tape on a horizontal bar 35 cm above the ground, with the suspension time set to 6 min. During the experiment, the mouse’s behavior was recorded using video surveillance, and the immobility time during the last 4 min was measured with a stopwatch. Immobility time, in seconds, was defined as the duration during which the mouse ceased struggling and remained completely still. After completing the experiment, the apparatus was sanitized using a 75% alcohol solution to ensure that no contamination occurred in following tests.

### Forced swim test

Mice were immersed in a transparent cylinder (diameter: 20 cm; height: 30 cm) containing approximately 20 cm of water maintained between 22 and 25 °C. Each trial lasted 6 min, during which the animal was allowed to swim freely. Behavioral activity was recorded using a video tracking system, and immobility during the final 4 min was quantified with a stopwatch. Immobility was defined as the period during which the mouse ceased active swimming and remained stationary. At the conclusion of each trial, the mouse was removed from the cylinder, gently dried with a towel, and provided adequate rest. The cylinder was subsequently cleaned to eliminate residual olfactory cues, thereby ensuring a standardized environment for subsequent trials.

### Drug and virus treatment

#### C3aR antagonist treatment

SB290157 (Calbiochem, Cat. No. SB290157) was dissolved in dimethyl sulfoxide (DMSO; 10 mg/ml stock) and diluted in 0.9% saline before use (final DMSO <1%). Mice received intraperitoneal injections of vehicle (saline with <1% DMSO) or SB290157 (1 mg/kg) 3 times weekly (every other day) during adolescent social isolation (PNDs 28 to 49). SB290157 has been demonstrated to penetrate the blood–brain barrier and achieve pharmacologically active concentrations in the central nervous system following systemic administration, as established in prior rodent studies [77–79].

#### Viral delivery

AAV9-CaMKIIα-hM3D(Gq)-mCherry or AAV9-CaMKIIα-mCherry (WZ Biosciences; 2.5 × 10^12^ vg/ml) were bilaterally injected into the DG at PND 28. The stereotaxic coordinates (from bregma) are anteroposterior (AP) −2.0 mm, mediolateral (ML) ±1.2 mm, and dorsoventral (DV) −2.2 mm. The injection volume was 300 nl/side at 50 nl/min. Mice recovered for 3 weeks before experiments. Viral expression was verified histologically; mice with off-target expression were excluded [80,81].

### Chemogenetic activation

Clozapine-*N*-oxide (CNO; Tocris Bioscience, Cat. No. 6329) was dissolved in DMSO and diluted in sterile 0.9% saline (final DMSO <1%) to prepare a fresh working solution on each experimental day. Mice received intraperitoneal injections of either CNO (5 mg/kg body weight) or vehicle (equal volume of 0.9% saline) 30 min prior to behavioral testing to coincide with peak CNO brain levels. Both hM3Dq-expressing mice and mCherry control mice received identical CNO treatments to control for any nonspecific effects of CNO itself. The 5 mg/kg dose was selected based on previous literature demonstrating robust designer receptors exclusively activated by designer drugs (DREADD) activation without off-target effects. Throughout the experimental period, all animals were monitored daily by trained personnel for signs of seizure activity, including convulsive behaviors (tonic-clonic seizures and myoclonic jerks), abnormal postures, stereotypies, or sudden death. No such events were observed in any experimental group [82–84].

### Immunofluorescence

Following adequate anesthesia with isoflurane, mice were transcardially perfused with pre-chilled 0.01 M phosphate-buffered saline (PBS) to remove blood, followed by perfusion with 4% paraformaldehyde (PFA) to fix tissues. Brains were subsequently extracted and immersed in 30% sucrose solution for cryoprotection until they sank to the bottom. After dehydration, tissues were embedded in optimal cutting temperature compound and rapidly frozen at −80 °C. For cryosectioning, samples were equilibrated at −20 °C and sectioned into 30-μm coronal slices using a Leica CM1950 cryostat. Sections were collected serially throughout the anterior–posterior extent of the hippocampus to ensure consistent and representative sampling across all animals. Sections were then transferred into cryoprotectant solution to preserve antigenicity and stored at low temperatures. For immunostaining, sections were rinsed 3 times with 0.01 M PBS to remove the cryoprotectant, followed by permeabilization in 0.5% Triton X-100 in PBS for 1 h. They were subsequently incubated in blocking solution (0.3% Triton X-100 and 5% normal donkey serum in 0.01 M PBS) at room temperature for 30 min. Sections were then incubated with primary antibodies at 4 °C overnight. After primary incubation, they were washed 3 times with 0.01 M PBS and exposed to secondary antibodies at room temperature in the dark for 2 h. Prior to mounting, nuclei were counterstained with 4′,6-diamidino-2-phenylindole. All antibodies used in this study are listed in Table S1.

### Confocal imaging, 3D reconstruction, and phagocytosis quantification

Synaptic imaging was performed using an ultrahigh-resolution confocal microscope (SPARC; Nikon Spatial Array Confocal) equipped with a 60× oil immersion objective. For synaptic marker analysis, the *z*-axis optical section thickness was set to 1 μm, and 3 consecutive optical slices were acquired to reliably distinguish pre- and postsynaptic signals. For microglial imaging, the same objective was employed with a *z*-axis step size of 0.5 μm, acquiring 60 consecutive sections to reconstruct the complete 3D microglial structure. A total of 12 to 14 microglia per mouse were selected for analysis, ensuring clear separation of ionized calcium-binding adapter molecule 1 (Iba-1), CD68, VGluT1, PSD-95, VGAT, and gephyrin signals. Image analysis was conducted using the Imaris software. The Spot detection function was applied to identify synaptic puncta positive for both pre- and postsynaptic markers. The number of pre- and postsynaptic puncta within each region of interest was quantified, and punctum density was subsequently calculated. Colocalization analyses of synaptic marker signals were performed to evaluate the structural integrity and spatial distribution of synaptic connections. Three-dimensional reconstructions were generated using Imaris, with the Surface rendering function employed to model the morphology of Iba-1-positive microglia and CD68-positive lysosomes, thereby providing comprehensive visualization of microglial structure and lysosomal organization. The Spot function was further used to detect synaptic puncta within Iba-1^+^ microglia and CD68^+^ lysosomes, enabling quantitative assessment of phagocytosed synapses. Subsequently, the count of engulfed synaptic puncta per individual microglial cell was determined.

### Bulk RNA-seq analysis

After ensuring sufficient isoflurane anesthesia, hippocampal samples were promptly isolated from mice, and RNA extraction was performed using TRIzol reagent. RNA concentration, purity, and integrity (RNA integrity number [RIN] ≥ 7.0) were assessed with a NanoDrop spectrophotometer and Agilent 2100 Bioanalyzer to ensure high sample quality. Only RNA samples meeting the criteria of RIN ≥ 7.0, concentration ≥ 50 ng/μl, and an OD_260_/OD_280_ ratio between 1.8 and 2.0 were retained for further analysis. RNA-seq libraries were prepared using the Illumina library construction kit, which included messenger RNA enrichment, fragmentation, complementary DNA synthesis by reverse transcription, end repair, and adapter ligation. The resulting libraries were polymerase chain reaction amplified and purified with magnetic beads to achieve uniform fragment distribution. Library concentration and fragment size distribution were evaluated using Qubit and Agilent 2100 Bioanalyzer. Sequencing was performed on the Illumina NovaSeq 6000 platform using a paired-end 150-bp (PE150) strategy to ensure sufficient sequencing depth for downstream analysis. Raw sequencing data were processed with Trimmomatic to remove adapter sequences and low-quality reads, and the resulting clean reads were quality-checked using FastQC. Clean data were aligned to the mouse reference genome (GRCm38) using HISAT2 or STAR, and gene quantification was carried out with featureCounts or HTSeq. Differential expression analysis was conducted with DESeq2 or edgeR, applying a threshold of |log_2_FC| ≥ 0.5 and adjusted *P* < 0.05. Finally, functional annotation was performed on differentially expressed genes. GO enrichment and KEGG pathway analyses were conducted using ClusterProfiler or DAVID, and results were visualized as GO bar plots and KEGG bubble charts to elucidate the biological functions and signaling pathways associated with the observed transcriptomic changes.

### Enzyme-linked immunosorbent assay

Hippocampal tissues were meticulously dissected from brains under 4 °C conditions and rapidly placed into chilled 0.01 M PBS solution. Using a homogenizer, tissues were processed at a 1:9 (g/ml) tissue–buffer ratio until uniform homogenization occurred. The resulting homogenate underwent centrifugation (4 °C, 12,000 rpm, 20 min), and the supernatant was collected for analysis. Protein content was assessed via a bicinchoninic acid (BCA) protein assay kit (P0012S, Beyotime). Quantified protein samples were then subjected to ELISA. Prior to use, the ELISA kit (Well-bio, Shanghai, China) was equilibrated at room temperature for 30 min. Samples were diluted 5-fold with the provided diluent and loaded into designated wells of the ELISA plate, which included wells for blanks, standards, samples, and distilled water. According to the manufacturer’s protocol, reagents were added in sequence, and the optical density was determined at 450 nm with a microplate reader (Bio-Rad xMark). A standard curve was generated by plotting absorbance values against known standard concentrations, and sample concentrations were calculated accordingly.

### Transmission electron microscopy

TEM was employed to examine the ultrastructure of mouse brain tissue. Following adequate anesthesia with isoflurane, mice were transcardially perfused with 0.01 M PBS to remove blood, after which a fixative solution containing 5% formaldehyde and 0.5% glutaraldehyde was slowly perfused through the brain for approximately 10 min. The hippocampal tissue was then rapidly excised, sectioned, and postfixed overnight in the same fixative. Subsequently, the tissue was rinsed 3 times with 0.01 M PBS and transferred to 1% osmium tetroxide for postfixation at 4 °C for 2 h. After postfixation, samples were dehydrated through a graded ethanol series (50% to 100%) and treated with anhydrous acetone to remove residual water. The tissue was infiltrated with a resin–acetone mixture, embedded in pure epoxy resin, and polymerized at room temperature for 48 h. Ultrathin sections (50 to 70 nm) were prepared using an ultramicrotome and sequentially stained with 2% uranyl acetate and 0.2% lead citrate to enhance contrast. Finally, sections were examined under a transmission electron microscope, and ultrastructural images were captured at appropriate accelerating voltages and magnifications.

### DiI staining

Mice were anesthetized with isoflurane, and after confirming an adequate depth of anesthesia, transcardial perfusion was performed with 1.5% PFA to fix the tissue. The brains were then removed, immersed in 1.5% PFA for an additional 1 h of postfixation, and subsequently transferred to 0.01 M PBS for storage. Coronal brain slices (100 μm thick) were prepared using a Leica vibratome and maintained in 0.01 M PBS to prevent desiccation. Sonicated DiI dye was carefully applied to axonal and circuit regions, and tissues were incubated for 12 h to allow for complete staining. Following the staining procedure, surplus dye and fixative were washed off using 0.01 M PBS, after which sections were preserved with antifade mounting medium. Finally, stained sections were imaged by fluorescence microscopy to visualize and assess the distribution of dendritic structures within neurons.

### Western blot

The hippocampal tissue of experimental mice was collected and lysed using radioimmunoprecipitation assay lysis buffer. Subsequently, the lysate underwent centrifugation at 13,000 rpm for 15 min at 4 °C, after which the supernatant was harvested. Protein levels were quantified via BCA assay. The protein samples were diluted with 5 times the volume of sodium dodecyl sulfate (SDS)–polyacrylamide gel electrophoresis sample buffer (Yeasen) and boiled at 95 °C. Then, proteins were separated using a 10% SDS–polyacrylamide gel. The proteins were transferred to a polyvinylidene fluoride membrane and incubated at room temperature with 5% nonfat milk for 2 h to block nonspecific binding sites. Primary antibody incubation was conducted overnight at 4 °C. Membranes were subsequently washed thrice with tris-buffered saline with Tween 20 (TBST) (10 min each) and then incubated with secondary antibody at ambient temperature for 1 h. The membrane was then washed 3 times with TBST for 10 min each. Protein detection was performed using an enhanced chemiluminescence reagent (BBI), and imaging was carried out using the E-blot imaging system.

### Nissl staining

Frozen brain sections (30 μm thick) were rinsed 3 times in 0.01 M PBS for 5 min each. The sections were subsequently stained with 0.5% Nissl solution (toluidine blue method, G1434, Solarbio) for 5 min. Following the staining procedure, tissue sections were processed through an ascending ethanol gradient (75%, 85%, 90%, 100%, and 100%) with 10-min intervals and then cleared using xylene for 30 min. Finally, sections were mounted with neutral resin and imaged using a scanning microscope (VS120, OLYMPUS).

### Microelectrode array

Following isoflurane anesthesia, mice underwent craniotomy for brain extraction. Extracted brains were promptly transferred into chilled artificial cerebrospinal fluid (ACSF) at 4 °C to ensure tissue preservation. Prior to use, ACSF was equilibrated with a gas mixture of 95% O_2_ and 5% CO_2_ for 30 to 60 min to stabilize the pH at 7.4. Throughout the experiment, the tissue was kept cooled and continuously oxygenated to maintain its physiological state. During sectioning, the brain was trimmed to isolate the target region and secured onto a sample tray. Ice blocks were positioned around the slicing chamber to maintain low temperature, and pre-chilled ACSF was continuously perfused with oxygenation. Coronal brain slices (300 μm thick) were prepared using a Leica VT1200S vibratome along the same anterior–posterior hippocampal axis. Electrophysiological recordings were acquired using the MaxOne MEA chip. Prior to recordings, the MEA surface was rinsed with distilled water, and brain slices were carefully positioned onto the central recording area, ensuring alignment of the target region (e.g., hippocampal DG) with the electrode array. Once the system was stabilized, the slice position was verified and documented under a microscope. A microporous filter membrane (2 × 4 mm) was placed over the slice and gently secured with a mesh to ensure optimal contact between the tissue and the electrode array. The perfusion system delivered pre-equilibrated ACSF (95% O_2_ and 5% CO_2_) at a constant flow rate to maintain slice viability. The mesh height was adjusted to stabilize the slice while preventing mechanical damage. Data acquisition commenced once electrophysiological signals reached a stable baseline.

### Stereotaxic microinjection

Viral injections and stereotaxic procedures were performed based on previously published methods. Briefly, isoflurane-anesthetized mice were mounted on a stereotactic instrument (RWD, USA). Bilateral viral vector administration into the hippocampal DG was achieved using a microinjection pump (Stoelting, USA). The stereotaxic coordinates for DG targeting were as follows: AP, −2.0 mm; ML, ±1.2 mm; and DV, −2.2 mm. Injections were performed at a rate of 50 nl/min. Upon completion, the pipette was left in place for 10 min to facilitate viral diffusion before being slowly withdrawn to minimize retrograde leakage. Following wound closure, mice were placed on a heating pad to recover and monitored closely for 3 d postoperatively. Injection sites were verified by immunofluorescence staining, and only animals with accurately targeted injections were included in subsequent experiments.

### Chemical genetics approach

Viruses (AAV9-CaMKIIα-hM3D(Gq)-mCherry or AAV9-CaMKIIα-mCherry) were injected into the DG region of the hippocampus in mice at postnatal day 28 (P28). Following viral delivery, mice were allocated to distinct housing environments according to their experimental groups. Three weeks after injection, neuronal activation was induced by intraperitoneal administration of CNO. CNO was dissolved in DMSO and subsequently diluted in physiological saline to a final dosage of 5 mg/kg. Behavioral testing commenced 30 min after CNO injection, based on established pharmacokinetic evidence demonstrating that CNO reaches effective concentrations in the brain within 15 to 30 min following intraperitoneal administration, with peak neuronal activation persisting for approximately 1 to 2 h [85,86]. Both hM3D(Gq)-expressing mice and mCherry control mice received CNO injections to control for any nonspecific effects of CNO itself. To validate the time course of neuronal activation, a separate cohort of mice was perfused 30 min after CNO administration (5 mg/kg, intraperitoneal) for c-Fos immunofluorescence analysis in the DG. Robust c-Fos expression was confirmed in hM3D(Gq)-expressing mice compared to mCherry controls, verifying effective neuronal activation at the time point of behavioral assessment (Fig. 2R). Injection sites were verified by immunofluorescence staining for mCherry expression, and only animals with accurately targeted viral expression restricted to the DG were included in subsequent analyses.

### Statistical analysis

Animals were randomly assigned to experimental groups, and the sample size (*n*) is indicated in the corresponding figure legends. Sample size determination was based on previously published studies. Statistical evaluations were performed with the GraphPad Prism 8 software. Data are shown as mean ± standard error of the mean. Distribution normality was evaluated via Kolmogorov–Smirnov and Shapiro–Wilk tests. For comparisons between 2 groups, a 2-tailed Student *t* test was applied. For multiple-group comparisons, 1-way or 2-way analysis of variance was performed, followed by Bonferroni or Tukey’s post hoc tests where appropriate. Statistical significance was set at *P* < 0.05, with significance levels indicated as follows: *P < 0.05, **P < 0.01, and ***P < 0.001. All data processing and statistical analyses were performed in a double-blind manner whenever feasible.

## Data Availability

The raw sequence data reported in this paper have been deposited in the Genome Sequence Archive (*Genomics, Proteomics & Bioinformatics* 2025) in the National Genomics Data Center (*Nucleic Acids Research* 2025), China National Center for Bioinformation/Beijing Institute of Genomics, Chinese Academy of Sciences (GSA: CRA032067), which are publicly accessible at https://ngdc.cncb.ac.cn/gsa.

## Supplementary Materials

Supplementary 1 Figs. S1 to S4 Figs. 1R to 3R Table S1
