# Supplementary material for: Maternal Stress Primes Adolescent Stress Susceptibility via Microglial Complement-Dependent Synaptic Phagocytosis
Source: Research (Wash D C). 2026 Aug 5;9:1367. doi: 10.34133/research.1367 (PMC13438034; doi:10.34133/research.1367)
Supplement: Supplementary file 1 [file research.1367.f1.zip › Figure-revised.docx]

**Fig.1R (A)** Representative western blot image of C3 and C3aR protein in the hippocampal DG region of female offspring. **(B)** The expression of C3/GAPDH in the hippocampal DG region of female offspring. **(C)** The expression of C3aR/GAPDH in the hippocampal DG region of female offspring. Each group consists of three mice, and three samples per mouse are analyzed. SI: Social Isolation; Quantitative data are expressed as the mean ± standard error of the mean (S.E.M). *P < 0.05, **P < 0.01, ***P < 0.001; ns, not significant. P values were determined by one-way ANOVA with Tukey’s post hoc test.

**Fig.2R (A)** Representative image of c-Fos immunofluorescence staining in the hippocampal DG region. **(B)** The number of c-Fos-positive cell in the hippocampal DG region. Data were collected from six mice per group, with three brain slices per mouse. Scale bar: 100μm. SI: Social Isolation; Quantitative data are expressed as the mean ± standard error of the mean (S.E.M). *P < 0.05, **P < 0.01, ***P < 0.001; ns, not significant. P values were determined by one-way ANOVA with Tukey’s post hoc test.

**5 μm**

**5 μm**

**Fig.3R (A)** Representative immunofluorescence images and corresponding 3D surface reconstructions of the complement protein C3 deposited on NeuN⁺ neurons in the hippocampal DG region. Scale bar: 5μm. **(B)** Representative immunofluorescence images and corresponding 3D surface reconstructions of complement protein C3 co-localized with the astrocyte marker GFAP in the hippocampal DG region. Scale bar: 5μm. **(C)** Quantification of the number of C3⁺ puncta per astrocyte in the hippocampal DG region. Each group consists of five mice, and three samples per mouse are analyzed. SI: Social Isolation; Quantitative data are expressed as the mean ± standard error of the mean (S.E.M). *P < 0.05, **P < 0.01, ***P < 0.001; ns, not significant. P values were determined by one-way ANOVA with Tukey’s post hoc test.
