# Supplementary material for: Maternal Stress Primes Adolescent Stress Susceptibility via Microglial Complement-Dependent Synaptic Phagocytosis
Source: Research (Wash D C). 2026 Aug 5;9:1367. doi: 10.34133/research.1367 (PMC13438034; doi:10.34133/research.1367)
Supplement: Supplementary file 1 [file research.1367.f1.zip › Table S1.docx]

| **Immunofluorescence Antibody** | | | | |
| --- | --- | --- | --- | --- |
| Antibody | Species | Manufacturer | Catalog Number | Dilution |
| Iba-1 | Guinea pig | Oasis | PGP049-01 | 1:500 |
| Iba-1 | Rabbit | Abcam | ab259971 | 1:1000 |
| CD68 | Rat | Bio-rad | MCA1957 | 1:500 |
| VGluT1 | Rabbit | Abcam | ab272913 | 1:500 |
| NeuN | Rabbit | Abways | CY5515 | 1:500 |
| PSD95 | Rabbit | Abcam | ab18258 | 1:500 |
| CamKIIα | Rabbit | Huabio | JM11-07 | 1:500 |
| GAD67 | Rat | Oasis | PRT084-01 | 1:500 |
| C3 | Mouse | Hycult | HM078 | 1:500 |
| C3aR | Mouse | Santa Cruz | sc-133172 | 1:500 |
| NeuN | Mouse | Sigma-Aldrich | MAB377 | 1:500 |
| PSD95 | Guinea pig | Synaptic Systems | 124308 | 1:1000 |
| VGAT | Guinea pig | Synaptic Systems | 131 005 | 1:1000 |
| c-Fos | Guinea pig | Synaptic Systems | 226308 | 1:1000 |
| Gephyrin | Mouse | Synaptic Systems | 147011 | 1:1000 |
|  |  |  |  |  |
| **WB Antibodies** | | | | |
| Antibody | Species | Manufacturer | Catalog Number | Dilution |
| VGluT1 | Rabbit | Abcam | ab272913 | 1:2000 |
| PSD95 | Rabbit | Abcam | ab259971 | 1:2000 |
| C3 | Mouse | Hycult | HM078 | 1:2000 |
| C3aR | Mouse | Santa Cruz | sc-133172 | 1:2000 |
| GAPDH | Rabbit | Abcam | ab272913 | 1:5000 |
| Bcl-2 | Rabbit | Abcam | ab182858 | 1:2000 |
| Bax | Rabbit | Abcam | ab32503 | 1:2000 |
| Caspase3 | Rabbit | Abcam | ab32351 | 1:2000 |
|  |  |  |  |  |
| **Immunofluorescence Secondary Antibodies** | | | | |
| Antibody | Species | Manufacturer | Catalog Number | Dilution |
| Guinea-Alexa Fluor 488 | Goat | Invitrogen | A11073 | 1:10000 |
| Rabbit-Alexa Fluor 568 | Goat | Invitrogen | A11036 | 1:10000 |
| Mouse-Alexa Fluor 568 | Goat | Invitrogen | A11031 | 1:10000 |
| Rabbit-Alexa Fluor 568 | Donkey | Invitrogen | A10042 | 1:10000 |
| Mouse-Alexa Fluor 568 | Donkey | Invitrogen | A10037 | 1:10000 |
| Mouse-Alexa Fluor 647 | Goat | Invitrogen | A21236 | 1:10000 |
| Rabbit-Alexa Fluor 647 | Goat | Invitrogen | A21245 | 1:10000 |
|  |  |  |  |  |
| **WB Secondary Antibodies** | | | | |
| Antibody | Species | Manufacturer | Catalog Number | Dilution |
| HRP-Anti-Rabbit IgG(H+L) | Goat | Proteintech | SA00001-2 | 1:5000 |
| HRP-Anti-Mouse IgG(H+L) | Goat | Proteintech | SA00001-1 | 1:5000 |
