# Supplementary material for: Maternal Stress Primes Adolescent Stress Susceptibility via Microglial Complement-Dependent Synaptic Phagocytosis
Source: Research (Wash D C). 2026 Aug 5;9:1367. doi: 10.34133/research.1367 (PMC13438034; doi:10.34133/research.1367)
Supplement: Supplementary file 1 [file research.1367.f1.zip › Supplemental Figure.doc]

**Supplementary Fig.1 Maternal stress did not alter stress sensitivity in female offspring during adolescence.**

**(A)**. Chronic unpredictable stress paradigm during pregnancy. **(B)** Representative traces of open-field test. **(C)** The total distance in the open field test (n = 12). **(D)** The time spent in the central zone in the open field test (n = 12). **(E)** Representative traces of elevated plus maze. **(F)** The time spent in the open-arms in the elevated plus maze test (n = 12). **(G)** The number of entries into the open arms in elevated plus maze test (n = 12). **(H)** The sucrose preference ratio in the sucrose preference test (n = 12). **(I)** The immobility time in the tail suspension test (n = 12). **(J)** The immobility time in the forced swim test (n = 12). SI: Social Isolation; Quantitative data are expressed as the mean ± standard error of the mean (S.E.M). *P < 0.05, **P < 0.01, ***P < 0.001; ns, not significant. P values were determined by one-way ANOVA with Tukey’s post hoc test. Source data and test statistics are provided as a Source Data file. See also Table S2.

**Supplementary Fig.2** **Neither maternal stress nor adolescent social isolation led to neuronal loss and apoptosis in the hippocampal region of male offspring.**

**(A)** Representative image of Nissl staining; Scale bar: 100μm. **(B)**. The number of intact neurons in the hippocampal CA1 region. **(C)** The number of intact neurons in the hippocampal CA3 region. **(D)** The number of intact neurons in the hippocampal DG region. Each group consisted of four mice, and three brain slices per mouse were analyzed. **(E)** Representative immunofluorescence image of the neuronal marker NeuN and the apoptosis marker Cleaved-caspase3 in the hippocampal CA1 region; Scale bar: 100μm. **(F)** Representative immunofluorescence image of the neuronal marker NeuN and the apoptosis marker Cleaved-caspase3 in the hippocampal DG region; Scale bar: 100μm. **(G)** Representative immunofluorescence image of the neuronal marker NeuN and the apoptosis marker Cleaved-caspase3 in the hippocampal CA3 region; Scale bar: 100μm. **(H)** The percentage of Cleave-caspase3^+^ NeuN^+^/NeuN^+^ cell in the hippocampal CA1 region. **(I)** The percentage of Cleave-caspase3^+^ NeuN^+^/NeuN^+^ cell in the hippocampal DG region. **(J)** The percentage of Cleave-caspase3^+^ NeuN^+^/NeuN^+^ cell in the hippocampal CA3 region. Each group consisted of three mice, and four brain slices were analyzed per mouse. **(K)** Representative western blot image of Bax, Bcl-2, and Caspase3 proteins in the hippocampal region. **(L)** The expression of Caspase3/GAPDH in the hippocampal region. **(M)** The expression of Bcl-2/Bax in the hippocampal region. Each group consisted of three mice, and three samples were analyzed per mouse. SI: Social Isolation; Quantitative data are expressed as the mean ± standard error of the mean (S.E.M). *P < 0.05, **P < 0.01, ***P < 0.001; ns, not significant. P values were determined by one-way ANOVA with Tukey’s post hoc test. Source data and test statistics are provided as a Source Data file. See also Table S2.

**Supplementary Fig.3 Neither maternal stress nor adolescent social isolation affected changes in neuronal activity in the hippocampal CA1 and CA3 regions of male offspring.**

**(A)** Representative image of c-Fos immunofluorescence staining in the hippocampal CA1 region. **(B)** Representative image of c-Fos immunofluorescence staining in the hippocampal CA3 region. **(C)** The number of c-Fos-positive cell in the hippocampal CA1 region. **(D)** The number of c-Fos-positive cell in the hippocampal CA3 region. Data were collected from five mice per group, with three brain slices per mouse. Data were collected from five mice per group, with three brain slices per mouse. SI: Social Isolation; Quantitative data are expressed as the mean ± standard error of the mean (S.E.M). *P < 0.05, **P < 0.01, ***P < 0.001; ns, not significant. P values were determined by one-way ANOVA with Tukey’s post hoc test. Source data and test statistics are provided as a Source Data file. See also Table S2.

**Supplementary Fig.4** **Neither maternal stress nor adolescent social isolation affected microglial phagocytosis of inhibitory synapses in the hippocampal DG region of male offspring.**

**(A)** Representative immunofluorescence image of microglial marker Iba-1, lysosomal marker CD68, and inhibitory presynaptic marker Gephyrin co-staining in the hippocampal DG region. Scale bar: 5μm.

**(B)** The number of Gephyrin^+^ inhibitory presynaptic puncta within CD68^+^ lysosomes in the microglial cells. **(C)** Representative immunofluorescence image of microglial marker Iba-1, lysosomal marker CD68, and inhibitory presynaptic marker VGAT co-staining. Scale bar: 5μm. **(D)** The number of VGAT ^+^ inhibitory postsynaptic puncta within CD68^+^ lysosomes in the microglial cells. Each group consisted of five mice, and three brain slices were analyzed per mouse. SI: Social Isolation; Quantitative data are expressed as the mean ± standard error of the mean (S.E.M). *P < 0.05, **P < 0.01, ***P < 0.001; ns, not significant. P values were determined by one-way ANOVA with Tukey’s post hoc test. Source data and test statistics are provided as a Source Data file. See also Table S2.
